# Novel active compounds and the anti-diabetic mechanism of mulberry leaves

**DOI:** 10.3389/fphar.2022.986931

**Published:** 2022-10-05

**Authors:** Qiuyue Lv, Jinrong Lin, Xinyan Wu, Huanhuan Pu, Yuwen Guan, Peigen Xiao, Chunnian He, Baoping Jiang

**Affiliations:** ^1^ Institute of Medicinal Plant Development, Chinese Academy of Medical Sciences, Peking Union Medical College, Beijing, China; ^2^ Key Laboratory of Bioactive Substances and Resources Utilization of Chinese Herbal Medicine, Ministry of Education, Beijing, China

**Keywords:** mulberry leaf, flavonoids, type 2 diabetes, network pharmacology, molecular docking

## Abstract

Mulberry (*Morus alba* L.) leaves have long been considered beneficial in traditional Chinese medicine to treat infectious and internal diseases. Recently studies have discovered that the mulberry leaf’s total flavonoids (MLF) display excellent hypoglycemia properties. However, the active ingredients and their molecular mechanisms are still uncharacterized. In this study, we explored the hypoglycemic effects of MLF and mulberry leaf polysaccharides (MLP) on ob/ob mice, an animal model of type 2 diabetes mellitus (T2DM), compared with *Ramulus Mori* (Sangzhi) alkaloid (RMA). Network pharmacology was employed to identify the potential available targets and active compounds of MLF and RMA against hyperglycemia. Molecular docking, an insulin-resistant cell model and qPCR were employed to verify the antidiabetic activity of the critical compounds and the gene expression profiles of the top molecular targets. Here, the results showed that MLF and MLP improved glucose uptake in insulin-resistant hepatocytes. MLF, MLP and RMA alleviated insulin resistance and glucose intolerance in ob/ob mice. Unlike MLF and MLP, RMA administration did not influence the accumulation of intrahepatic lipids. Network pharmacology analysis revealed that morusin, kuwanon C and morusyunnansin L are the main active compounds of MLF and that they amend insulin resistance and glycemia *via* the PI3K- Akt signaling pathway, lipid and atherosclerosis pathways, and the AGE-RAGE signaling pathway. Moreover, 1-deoxynojirimycin (DNJ), fagomine (FA), and N-methyl-1-deoxynojirimycin are the primary active ingredients of RMA and target carbohydrate metabolism and regulate alpha-glucosidase activity to produce a potent anti-diabetic effect. The molecular docking results indicated that morusin, kuwanon C and morusyunnansin L are the critical bioactive compounds of MLF. They had high affinities with the key targets adenosine A1 receptor (ADORA1), AKT serine/threonine kinase 1 (AKT1), peroxisome proliferator-activated receptor gamma (PPARγ), and glycogen synthase kinase 3 beta (GSK3β), which play crucial roles in the MLF-mediated glucose-lowering effect. Additionally, morusin plays a role in amending insulin resistance of hepatocytes by repressing the expression of the *ADORA1* and *PPARG* genes. Our results shed light on the mechanism behind the glucose-lowering effects of MLF, suggesting that morusin, kuwanon C, and morusyunnansin L might be promising drug leads for the management of T2DM.

## Introduction

Type 2 diabetes mellitus (T2DM) is a chronic metabolic disease that may lead to multiple complications, such as cardiovascular, renal and ophthalmic complications (Ali et al., 2022). T2DM is relatively heterogeneous and very complex, involving multiple pathophysiological mechanisms that affect the pancreas and metabolic organs, making effective treatment very challenging (Demir et al., 2021).

Adenosine A1 receptor (ADORA1) is known to inhibit adenylate cyclase and play a role in regulating cell metabolism and gene transcription. Previous studies have shown that ADORA1 plays a vital role in carcinogenesis and is an important drug target in tumors (Liu et al., 2020a; Pan et al., 2021). Moreover, a recent study showed that ADORA1 involved in maintaining glucose homeostasis and regulating glucagon secretion as a G-protein-coupled receptor (Cheng et al., 2000). Meanwhile, activation of ADORA1 signaling in peripheral tissues facilitates high-fat diet-induced obesity. Specific inhibition of ADORA1 in the liver helps prevent body weight gain and alleviate hepatic steatosis, suggesting that ADORA1 might be a promising drug target for treating diabetes and obesity (Hong et al., 2019). Peroxisome proliferator-activated receptor gamma (PPARγ), a known target for thiazolidinediones, belongs to the nuclear receptor family. Activation of PPARγ results in increased insulin sensitivity in skeletal muscle and liver and improves the secretory profile of adipose tissue, favoring the release of insulin-sensitizing adipokines, such as adiponectin, and reducing inflammatory cytokines (Skat-Rordam et al., 2019; Wang et al., 2020a). However, thiazolidinediones cause adverse effects such as weight gain, fluid retention, bone fractures, and congestive heart failure, which impose a huge health burden (Kahn and McGraw, 2010). Interestingly, full and partial activation and antagonism of PPARγ can all improve insulin sensitivity (Ahmadian et al., 2013). Therefore, discovering novel selective modulators of PPARγ that evoke fewer side effects while possessing insulin-sensitizing potential is a vital goal.

Natural products derived from medicinal plants provide multiple health benefits (Al-Ishaq et al., 2019). Over the past 20 years, scientific attention has been given to natural compounds, that play pivotal roles in drug or lead discovery, especially for infectious diseases, diabetes, and cardiovascular disease (Ong and Khoo, 2000; Atanasov et al., 2021). Flavonoids are a group of polyphenolic compounds that are widely distributed in plants (Cao et al., 2019) and display various positive health effects on metabolic disorders. Studies have shown that flavonoid intake may decrease the risk of developing T2DM (Liu et al., 2014; investigators, 2015) by regulating targeted cellular signaling networks related to insulin secretion, glucose metabolism, and glucose transport in pancreatic β-cells, hepatocytes, skeletal myofibers, and adipocytes (Hussain et al., 2020). Therefore, developing and utilizing flavonoids are essential for the therapy and prevention of metabolic disorders.

Mulberry (*Morus alba* L.) is a plant belonging to the family *Moraceae* and the genus *Morus* (Lee et al., 2020). *Ramulus Mori* (Sangzhi) alkaloid (RMA), a group of effective polyhydroxy alkaloids derived from *Ramulus Mori* (Liu S. et al., 2019), is a novel inhibitor of α-glucosidase that the China National Medical Products Administration has approved for the treatment of T2DM (Liu et al., 2021). Therefore, mulberry leaves have been evaluated and have been found to exhibit excellent hypoglycemic activity and reduce inflammation and insulin resistance in T2DM (Tian et al., 2019; Li et al., 2020; Meng et al., 2020). Mulberry leaf flavonoids (MLF), polysaccharides (MLP) and alkaloids are the main functional components of mulberry leaves with various biological activities, such as antioxidation, hypolipidemia and hypoglycemia (Meng et al., 2020; Zhong et al., 2020). MLF ameliorates skeletal muscle insulin resistance (Meng et al., 2020), reduces the accumulation of lipids and hepatic steatosis, and whitens brown fat in diet- or gene deficiency-induced obese mice (Zhong et al., 2020). MLP effectively normalizes hepatic glucose metabolism and insulin signaling and mitigates oxidative stress in the livers of rats with T2DM induced by high fat diet and streptozotocin (Ren et al., 2015). RMA, as an inhibitor of α-glucosidase, mainly acts on the gut and delays the intestinal digestion of carbohydrates (Li et al., 2016). Hence, we employed ob/ob mice to compare the glucose -lowering effects of MLF, MLP and RMA and performed network pharmacology analysis to discover their potential active compounds and mechanisms, verifying the findings in human hepatocytes. A total of 29 flavonoids of mulberry leaf and 4 alkaloids of *Ramulus Mori* were collected from the TCMSP database and published literature for network analysis. Our results indicated that 1-deoxynojirimycin (DNJ), fagomine (FA), and N-methyl-1-deoxynojirimycin are the primary active compounds of RMA and target maltase-glucoamylase (MGAM) and sucrase-isomaltase (SI) proteins to lower glucose. Meanwhile, morusin, kuwanon C and morusyunnansin L are probably the important ingredients of MLF in hypoglycemia, which may function by regulating key targets, including ADORA1, AKT serine/threonine kinase 1 (AKT1), PPARγ and glycogen synthase kinase-3 beta (GSK3β).

## Methods

### Preparation of the crude extract of mulberry leaf

Mulberry (*Morus alba* L.) leaves were purchased from Beijing Tong Ren Tang Co., Ltd. (Beijing, China). The crude extract of mulberry leaf (MLE) was prepared with the following procedures. Approximately 100 g of mulberry leaves was refluxed with 1,400 ml water for 1 h. The filtrate was collected by filtration using a Buchner funnel and evaporated to obtain crude extracts (17.92 g).

### Preparation and quality control of mulberry leaf extract flavonoids

The MLF was prepared with the following procedures. One kilogram of mulberry leaves was refluxed with 60% ethanol (1:10, w/v) for 1 h, and then the filtrate was collected. A further 10,000 ml of 60% ethanol was added to the drug residue and refluxed for another 1 h. All the filtrates were collected and decompressed to concentrate. Then, the alcohol extract was purified on an AB-8 macroporous adsorption resin column (Shanghai Macklin Biochemical Co., Ltd., Beijing, China), and the elution solvent was a water-ethanol system (0%, 20%, and 70%). Finally, the eluent of 70% ethanol was collected and then concentrated to dryness.

**FIGURE 1:**
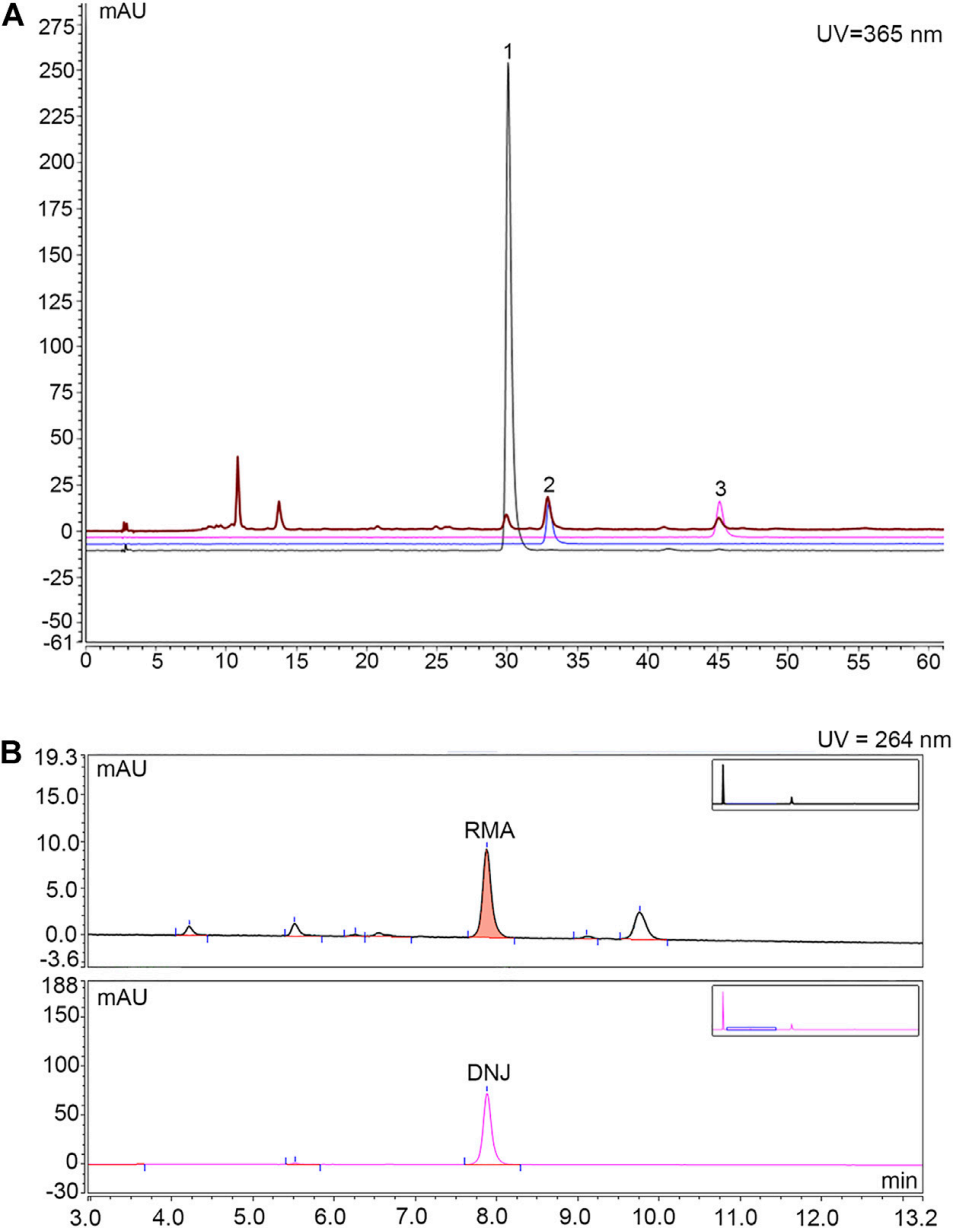
HPLC chromatogram for determining the content of rutin, isoquercitrin and astragalin in MLF and the content of DNJ in RMA. **(A)** Determination of rutin, isoquercitrin and astragalin contents in MLFs by HPLC analysis. The black, blue and purple lines represent rutin, isoquercetin, and astragalin, respectively. The brown line represents MLF. **(B)** Determination of DNJ content in RMA by HPLC analysis. The upper half represents RMA, whereas the lower half represents DNJ.

The main ingredients rutin, isoquercitrin, and astragalin in MLF were confirmed using an Agilent 1,260 liquid chromatography system (Meng et al., 2020). In brief, 15 μl MLF was injected into the apparatus with an autosampler. Chromatographic separation was performed using an Agilent C18 column (4.6 × 250 mm, 5 μm) with a flow rate of 1.0 ml/min. The mobile phases were A-0.1% (v/v) formic acid in water and B-acetonitrile. The gradient elution conditions are shown in Supplementary Table S1. The column temperature was 30°C, with a detection wavelength of 365 nm. The rutin, isoquercitrin and astragalin contents in MLF were 0.4954%, 0.8826%, and 0.3638%, respectively (Figure 1A; Table 1).

**TABLE 1:** Determination of rutin, isoquercitrin and astragalin contents in MLFs by HPLC analysis.

Serial number	Compound	Retentiontime (min)	Relative peak area	Concentration (mg/ml)	Content (%)
1	Rutin	29.920	3.4953	0.01392	0.4954
2	Isoquercitrin	32.860	9.0638	0.02480	0.8826
3	Astragalin	45.043	3.2860	0.02045	0.3638

### Estimation of the polysaccharide content in MLP

MLP was purchased from Shanghai Yuanye Bio-Technology Co., Ltd. (Shanghai, China), and the polysaccharide content in MLP was measured using the phenol‒sulfuric acid method (Dubios et al., 1956). According to the glucose standard curve (Supplementary Figure S2), the polysaccharide content of MLP was calculated to be 78.28%.

### Determination of the DNJ content in RMA

RMA was purchased from Beijing Wehandbio Co., Ltd. (Beijing, China), and the DNJ content was determined using high-performance liquid chromatography (HPLC) (Piao et al., 2018; Ma et al., 2019). The following analysis conditions were used: column, Agilent C18 column (4.6 × 250 mm, 5 μm); mobile phase, A-acetonitrile, B-75 mmol/L sodium citrate (pH = 4.21); flow rate, 1.5 ml/min; column temperature, 32°C; UV detector wavelength, 264 nm; and the injection volume, 10 µl. The gradient elution conditions are shown in Supplementary Table S2. The DNJ content in RMA was 9.58% (Figure 1B).

### Cell culture and glucose uptake experiment

The human normal liver L02 cell line was kindly provided by Dr. Jiyan Zhang (Academy of Military Sciences, Beijing, China). The insulin resistance cell model was established according to our previous method (Lv et al., 2019). Briefly, the drugs and extracts were first dissolved in DMSO and diluted with 1640 RPMI medium to appropriate experimental concentrations for cell exposure experiments. The final concentration of DMSO was less than 0.1%. Cells were seeded in a 96-well plate containing 1,640 medium supplemented with 10% fetal bovine serum (FBS, Gibco), 100 U/ml penicillin and 1% streptomycin (Gibco). Cells were cultured at 37 °C in a humidified atmosphere containing 5% CO_2_. Twenty-four hours after seeding, the cell medium was changed to 1,640 containing 2% FBS and 25 μmol/L lithocholic acid (LCA) for 24 h to induce insulin resistance. Next, different concentrations of drugs (0.5, 1, 2 mg/ml MLE; 25, 50, 100 mg/L MLF; 25, 50, 100 μmol/L DNJ; 62.5, 125, 250 mg/L MLP) or 25 μmol/L metformin (Met) were added and incubated for 24 h. After this incubation, cellular glucose uptake was examined using fluorescent 2-deoxy-2-[(7-nitro-2,1,3-benzoxadiazol-4-yl) amino]-D-glucose (2-NBDG, Invitrogen). Cells were washed with PBS and incubated with 100 μmol/L 2-NBDG supplemented with 1 × 10^-7^ mol/L insulin at 37 °C with 5% CO_2_ for 30 min. After the treatment, the cells were washed with PBS, and fresh PBS was added to each well (100 μL per well). The fluorescence was detected using a fluorescence microplate reader (excitation wavelength 488 nm, emission wavelength 520 nm).

### Cell viability assay

Cell viability was detected by MTT assay (Zhang et al., 2017). The protocol of drug administration was the same as that in the experiments described above. Briefly, 24 h after drug treatment, the culture medium was removed, and 100 μl of 1,640 medium containing 0.5 mg/ml MTT was added, followed by incubation at 37°C in a humidified atmosphere of 5% CO_2_. Four hours later, the culture medium was completely removed from each well, and 150 μl DMSO was used to dissolve the insoluble formazan crystals. The absorbance of the solvate of each well was detected by a microplate reader at 570 nm.

The cell glucose uptake rate (%) was calculated according to the following formula:

$$Cellular\;glucose\;uptake\;rate=\frac{\left[OD\;\left(Drug\;group\;cellular\;glucose\;uptake\right)÷OD\left(Drug\;group\;cell\;viability\right)\right]}{\left[OD\left(Control\;group\;cellular\;glucose\;uptake\right)÷OD\;\left(Control\;group\;cell\;viability\right)\right]}\times 100\%$$

### Cellular glucose consumption assessment

The cell culture conditions and reagents were the same as described above. L02 cells were cultured in 96-well plates and divided into six groups: control (Con), model (Mod), 1.25, 2.5, 5, and 10 μmol/L morusin. Cells in the Mod and drug groups were treated with 25 μmol/L LCA for 24 h. Then, cells in the drug groups were treated with morusin at different concentrations (1.25, 2.5, 5, and 10 μmol/L) for 24 h. Twenty-4 hours later, the culture medium was removed, and 100 μl of 1,640 medium was added. Cells were then incubated at 37°C for 6 h in a CO_2_ incubator. Subsequently, 5 μl of the supernatant from each well or different concentrations of the standard glucose solution were added to 100 μl of working solution. The absorbance was determined at 550 nm after incubation for 20 min at 37°C. The glucose consumption was calculated by subtracting the glucose concentration of blank wells from that of cell-plated wells. After aspirating the supernatant to detect glucose consumption, 20 μl MTT was added to each well, and the cells were then incubated at 37°C for 4 h. Cell survival was then detected through an MTT assay.

### Animals and treatments

Five-week-old (B6/JGpt-LepemICd25/Gpt, Leptinmut/mut) ob/ob mice and their leptinwt/wt (WT) littermates (GemPhamatech Co.,Ltd. Jiangsu, China) were housed under a 12 h light/dark cycle with free access to food and water. The animal experimental project was reviewed and approved by the Guidelines and Policies for Animal Surgery under the approval of the Chinese Academy of Medical Sciences and Peking Union Medical College, Beijing, China (approval No: SLXD-20200827001), and was approved by the Institutional Animal Use and Care Committee. After 7 days of acclimatization, the ob/ob mice were randomly divided into 4 groups containing 7 mice: the ob/ob, RMA, MLF, and MLP groups. Mice in the WT and ob/ob groups were fed a standard chow diet, while MLF and MLP mice received standard chow containing 1% (w/w) MLF or MLP, respectively. Mice in the RMA group were administered RMA by gavage (50 mg/kg). At 20 weeks of age, all mice were fasted for 12 h and terminally anesthetized with 200 mg/kg tribromoethanol. The blood was collected and centrifuged at 3,000 rpm for 10 min, and the serum was used to determine serum biomarkers. The livers were fixed in 4% paraformaldehyde or quickly frozen in liquid nitrogen and stored at −80°C for subsequent analysis.

### Oral glucose tolerance test and insulin tolerance test

At the 13th week, oral glucose tolerance tests (OGTTs) were performed on 12 h fasted mice administered a glucose solution (2 g/kg). In the 14th week, all mice were fasted for 4 h before the insulin tolerance tests (ITTs) were carried out, in which the mice were intraperitoneally injected with recombinant human insulin (0.75 U/kg, provided by Novo Nordisk). In the OGTTs and ITTs, the blood was taken from the tail vein, and blood glucose levels were detected at 0, 30, 60, 90 and 120 min using a glucometer and test strips after the glucose and insulin were given to the mice.

### Blood biochemical analysis

Serum glucose levels were measured by Beckman Coulter AU480 Automatic Biochemistry device using a glucose kit (ZHONGSHENG BEIKONG BIO-TECHNOLOGY AND SCIENCE, INC.). Insulin contents in the serum were detected using a mouse insulin ELISA kit (Beijing Sino-UK Institute of Biological Technology) according to the manufacturer’s directions.

Homeostasis model assessment-insulin resistance (HOMA-IR) and homeostasis model assessment-insulin sensitive index (HOMA-ISI) were used to evaluate the insulin resistance from basal glucose and insulin. The HOMA-IR and HOMA-ISI indices were calculated using the following formulas:

HOMA−IR=[fasting glucose (mmol/L)×fasting insulin (μU/mL)]/22.5

HOMA−ISI=1/[fasting glucose(mmol/L)×fasting insulin(μU/mL)]

### Hematoxylin-eosin staining

The liver samples were fixed in 4% formaldehyde, dehydrated, embedded in paraffin, and sectioned (5 μm). For histopathological evaluation, the paraffin-embedded liver sections were stained with hematoxylin and eosin (H&E).

### Compound target prediction and screening of disease targets

The flavonoids and alkaloids of mulberry leaves and *Ramulus Mori* were obtained from the Traditional Chinese Medicine Systems Pharmacology Database (TCMSP, http://tcmspw.com/tcmsp.php) (Ru et al., 2014) and published literature (Chen et al., 2000; Yang, 2010; Chen, 2014; Yang et al., 2015; Li, 2017). The SwissTargetPrediction database (http://www.swisstargetprediction.ch) (Daina et al., 2019) was utilized to predict the potential targets of the active molecules in MLF and RMA. *Homo sapiens* was selected as the target organism. The keyword “Type 2 diabetes” was used to collect potential genes. The T2DM-associated targets were acquired from the DrugBank database (https://go.drugbank.com/drugs) (Law et al., 2014), the Therapeutic Target Database (TTD, http://bid.nus.edu.sg/group/cjttd/) (Wang et al., 2020b), the Online Mendelian Inheritance in Man database (OMIM, https://omim.org/) (Amberger et al., 2015) and the human gene database (GeneCards, https://www.genecards.org/) (Stelzer et al., 2016). The targets of T2DM and predicted compound targets were verified using the UniProt database (https://www.uniprot.org/) (The UniProt, 2017), which was also employed to obtain the protein and gene names.

### Network construction

The overlapping genes between compounds and T2DM target genes were identified and visualized using Venn diagrams. The overlapping genes and their corresponding active compounds were imported into Cytoscape 3.7.1 software to construct a compound anti-diabetes target network. Furthermore, the Network Analyzer plug-in was utilized to analyze the topological parameters associated with the target degree. The degree value represents the number of nodes connected by a node. In the network, the size of the nodes represents the degree value, so the larger the node in the network is, the higher the degree value of the node.

### Construction of the PPI network

The targets of MLF and RMA for the treatment of T2DM were imported into the STRING database (https://www.string-db.org/) (Szklarczyk et al., 2019) to construct a protein‒protein interaction (PPI) network and analyze the functional interactions between proteins. Analysis was carried out with *Homo sapiens* as the organism option and with medium confidence greater than 0.4. The visualization process was performed using Cytoscape (Version 3.7.1), and the MCODE plugin in Cytoscape was used to detect clusters in the PPI network (Liu et al., 2020b). The parameters were as follows: degree cutoff ≥2, K-core ≥ 4, node score cutoff ≥0.2, and max depth = 100.

### GO and KEGG pathway enrichment analysis

GO (Gene Ontology) function and KEGG (Kyoto Encyclopedia of Genes and Genomes) pathway enrichment analyses were conducted to explore the core mechanism and pathway of MLF and RMA anti-diabetes in the Metascape database (http://metascape.org) (Zhou et al., 2019). We searched the gene symbols of common targets in the Metascape database by limiting the species to “*Homo sapiens*”, and setting the minimum overlap as 3 and the cutoff *p* value as 0.01 for enrichment analysis, including the GO (biological processes, cellular component, and molecular function) and KEGG pathways. The bubble charts were generated by the online bioinformatics tool (http://www.bioinformatics.com.cn/), and the target-pathway network was constructed using Cytoscape 3.7.1 software.

### Molecular docking

Classic molecular dynamics were used to analyze the interactions between compounds and target proteins using AutoDocTools-1.5.6, PyMOL-1.7.2.1, and Discovery Studio-2020 to elucidate the mechanism of the antidiabetic activity of these compounds (Liu et al., 2020b). Compound 3D structures were drawn using ChemDraw 20.0 and Chem3D 20.0 software. Crystal structures of target proteins were obtained from the RCSB Protein Data Bank (PDB, https://www.rcsb.org/) (Berman et al., 2000). AutoDockTools-1.5.6 and Discovery Studio-2020 were used to prepare the cocrystalized ligands split from the receptors and the active pocket of each target. The molecular docking simulations and free binding energy calculations were performed using AutoDock Vina-1.1.2. The binding interactions in the protein‒ligand complex were analyzed and visualized using Discovery Studio-2020 software and PyMOL-1.7.2.1.

### Western blot

Protein was extracted from livers using RIPA lysis buffer containing protease/phosphatase inhibitor cocktail (Beyotime Biotechnology). Antibodies against p-AKT (phospho Ser473, ab66138, Abcam), AKT (#4691, Cell Signaling Technology), p-GSK3β (phospho Ser9, #9322, Cell Signaling Technology), and GSK3β (#12456, Cell Signaling Technology) were used.

### Cell preparation

Cells in logarithmic-growth phase were inoculated in four 60 mm culture dishes and divided into four groups: Con group, Mod group and two drug groups. When the available space in the cell culture vessel reached 80% confluency, cells in the Mod and drug groups were treated with 25 μmol/L LCA for 24 h. Subsequently, cells in the drug groups were administered morusin at final concentrations of 2.5 and 5 μmol/L for 24 h. The cells were then harvested following insulin (1 × 10^−7^ mol/L) stimulation for 30 min.

### Real-time polymerase chain reaction analysis

Total RNA was isolated from liver and cells from the previous step using a total RNA extraction kit (RNAiso Plus, TaKaRa) according to the manufacturer’s instructions. First-strand cDNA was synthesized from total RNA using PrimeScript^TM^ RT Master Mix (TaKaRa). Real-time PCR was performed with a total volume of 20 μl, which contained 2 μl of cDNA, 0.4 μl of each 10 μM forward and reverse primer, 5.2 ml of ddH2O and 10 μl of 2×PerfectStart® Green qPCR SuperMix (TransGen Biotech) on a CFX96^TM^ Real-Time PCR Detection System. PCR amplification was performed using cycling conditions of 94°C for 30 s, followed by 45 cycles of 94°C for 5 s and 60°C for 30 s. Relative gene expression changes were measured by the comparative Ct method, X = 2^-△△Ct^ (Dong et al., 2022), using GAPDH as our housekeeping internal control gene. The primers used for qPCR are listed in Supplementary Table S3.

### Statistical analysis

GraphPad Prism 8.0 software was utilized for all data analyses. All values are presented as the mean ± standard error of the mean. Multiple groups or treatments were compared using one-way analysis of variance (ANOVA). Post-ANOVA comparisons were made using Dunnett’s correction. Differences were considered significant when *p* < 0.05.

## Results

### MLE, MLF, DNJ and MLP attenuated LCA-induced insulin resistance *in vitro*

**FIGURE 2:**
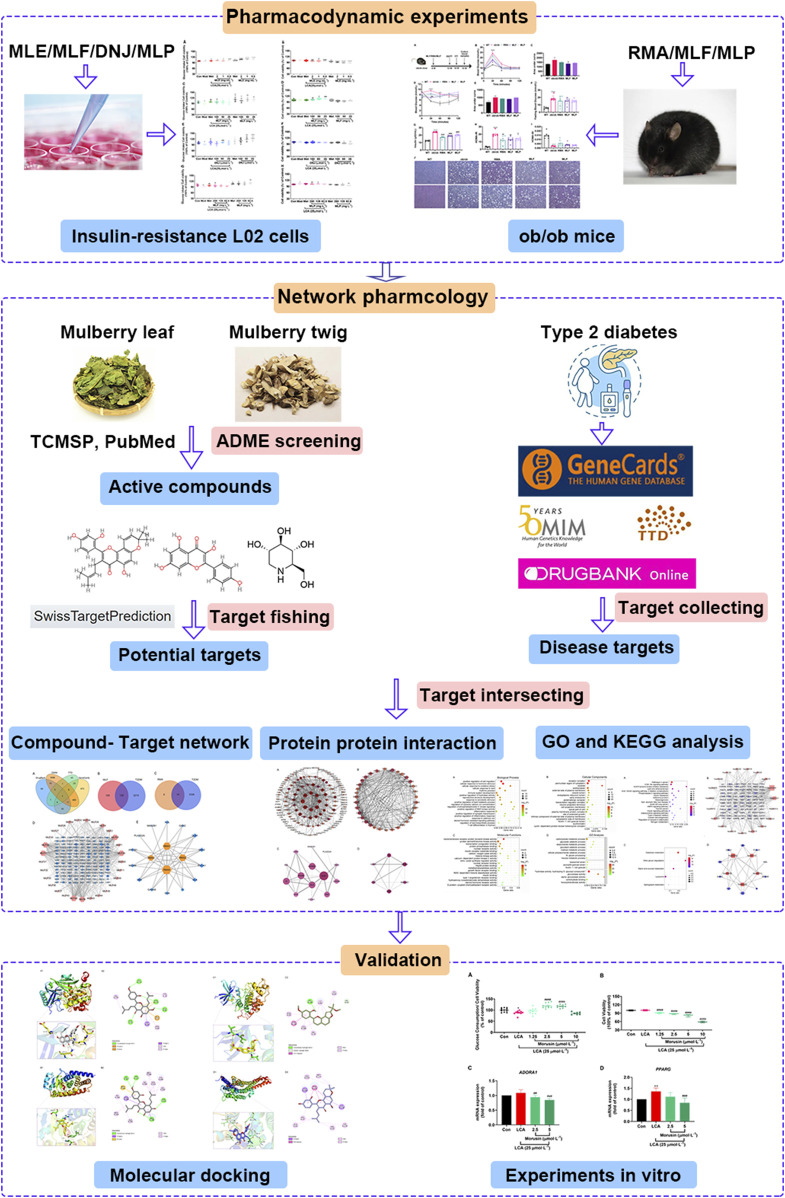
Integrated workflow for elucidating the active compounds and the underlying hypoglycemic mechanism of mulberry leaves.

The flow chart of this study is displayed in Figure 2. Our previous study established an insulin-resistant cell model using LCA to verify it using antidiabetic drugs and to screen plant ingredients (Lv et al., 2019). Our results demonstrated that cellular glucose uptake in the Mod group was lower than that in the Con group (*p* < 0.05). At the same time, 25 μmol/L Met treatment reversed this effect of LCA (*p* < 0.05), suggesting that the insulin-resistant model was successfully constructed and can be used to screen hypoglycemic compounds.

**FIGURE 3:**
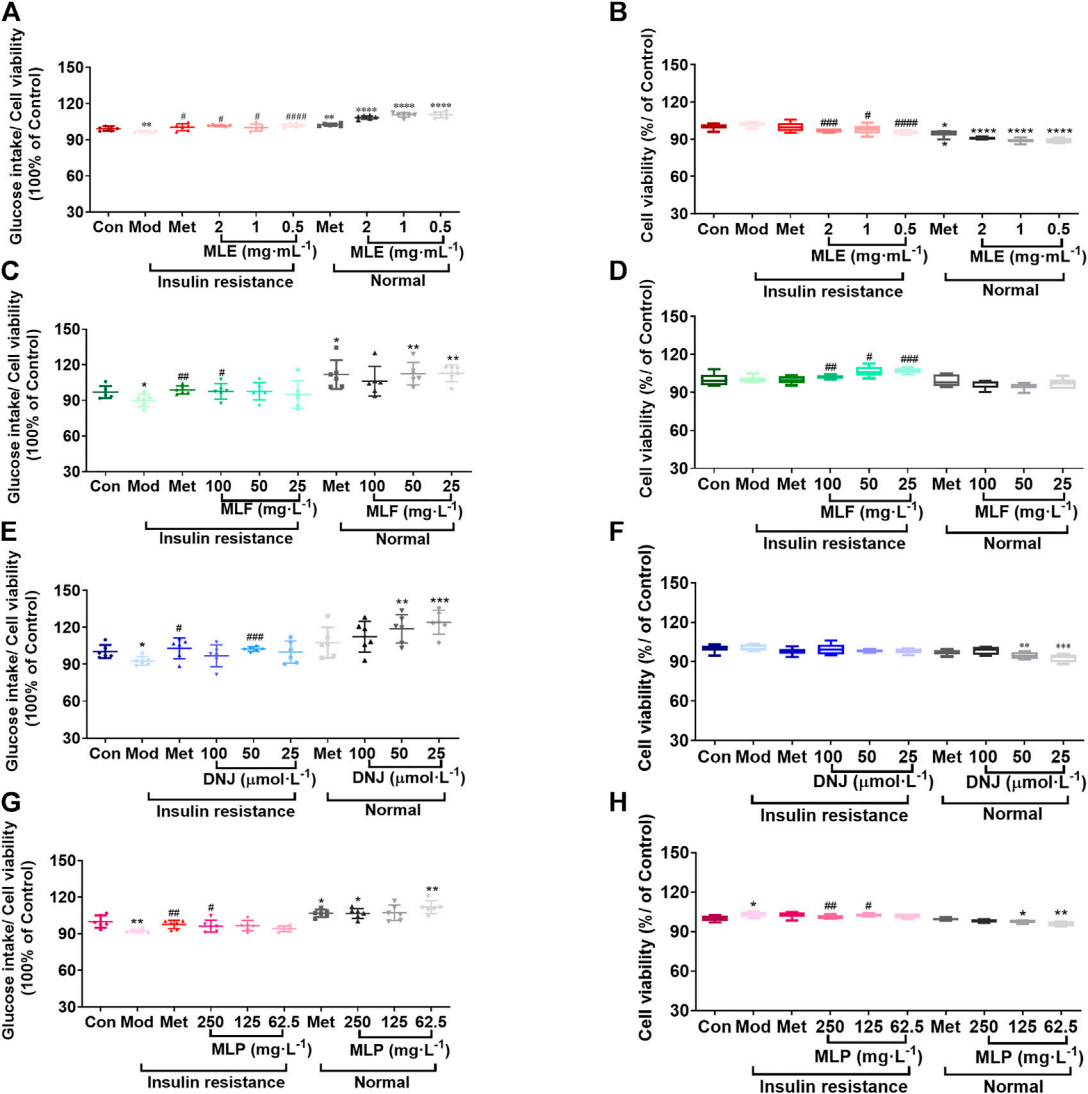
MLE, MLF, DNJ, and MLP treatment promotes glucose uptake in insulin-resistant L02 cells. Glucose intake levels in human L02 hepatocytes treated with MLE **(A)**, MLF **(C)**, DNJ **(E)** and MLP **(G)** at different doses. Viability of L02 hepatocytes treated with MLE **(B)**, MLF **(D)**, DNJ **(F)** and MLP **(H)** at different doses. *p < 0.05, **p < 0.01, ***p < 0.001, ****p < 0.0001, compared with Con group; ^#^ *p* < 0.05, ^##^ *p* < 0.01, ^###^ *p* < 0.001, ^####^ *p* < 0.0001, compared with Mod group.

Subsequently, we assessed the effects of MLE, MLF, DNJ, and MLP on cellular glucose uptake at different concentrations. The results showed that MLE at all treatment concentrations (0.5, 1 and 2 mg/ml) markedly increased glucose uptake in the presence or absence of LCA (Figure 3A). Compared with the Mod group, the 100 mg/L MLF, 50 μmol/L DNJ, and 250 mg/L MLP treatments all significantly alleviated cell insulin resistance induced by LCA. Treatment of L02 hepatocytes with MLF, DNJ and MLP also increased cellular glucose uptake stimulated by insulin (Figure 3). The MTT assay results showed that cell viability was obviously affected by MLE, MLF, DNJ or MLP treatment at the indicated concentrations (Figure 3). These results suggested the antidiabetic activity of MLF, DNJ and MLP in amending insulin-resistant hepatocytes.

### RMA, MLF and MLP alleviate glucose tolerance and insulin resistance in ob/ob mice

To compare the hypoglycemic effects of RMA, MLF and MLP, we fed ob/ob mice standard chow containing 1% MLF and 1% MLP for 14 weeks. Mice in the RMA group were administered RMA (50 mg/kg) by gavage. Compared with their lean counterparts, ob/ob mice exhibited marked increases in water intake, food intake, body weight, and food utility, which were not affected by RMA, MLF, or MLP treatments (Supplementary Figure S1).

**FIGURE 4:**
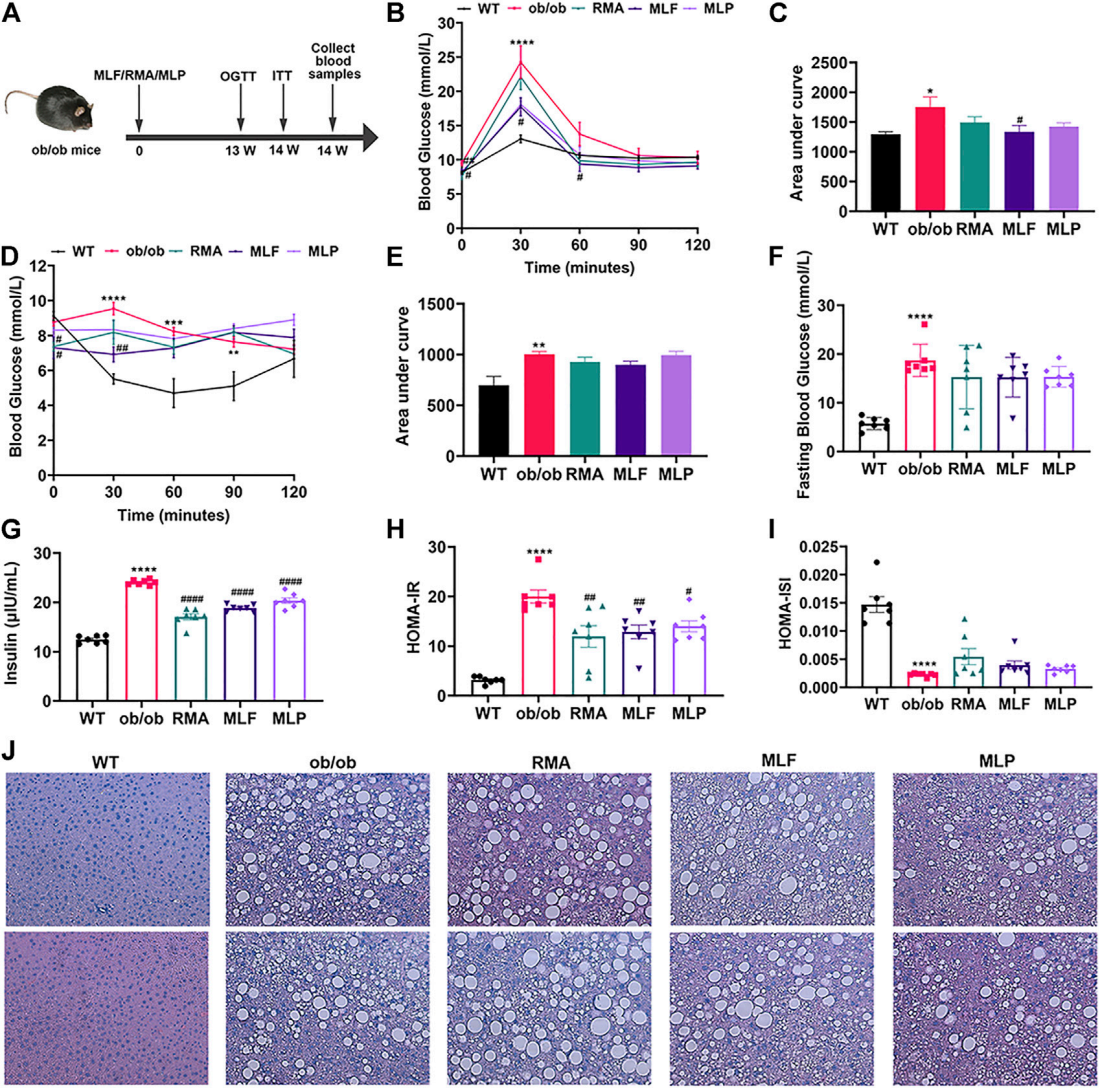
MLF and MLP treatment improved metabolic parameters of glucose tolerance and insulin resistance, and alleviated hepatic steatosis in ob/ob mice. **(A)** Experimental scheme of the ob/ob mouse protocol. **(B)** OGTT (oral glucose tolerance tests) and **(C)** AUC of the OGTT on the 13th week of treatment in ob/ob mice. **(D)** ITT (insulin tolerance tests) and **(E)** AUC of the ITT on the 14th week of treatment in ob/ob mice. **(F)** Fasting blood glucose in serum. **(G)** Serum insulin levels. **(H)** *HOMA*-*IR* index. **(I)** *HOMA*-*ISI* index. **(J)** Representative pictures of liver stained with H&E (magnification: ×40). *p < 0.05, **p < 0.01, ***p < 0.001, ****p < 0.0001, compared with WT group; ^#^ *p* < 0.05, ^##^ *p* < 0.01, ^###^ *p* < 0.001, ^####^ *p* < 0.0001, compared with the ob/ob group.

The OGTTs were performed on the 13th week of drug treatments, and the results revealed that ob/ob mice exhibited higher glycemic values following acute oral glucose than lean WT mice (Figure 4B). MLF significantly decreased blood glucose levels at 30 and 60 min after the glucose load in ob/ob mice, consistent with the area under the curve (AUC)-OGTT (Figures 4B,C). RMA and MLP treatments for 13 weeks significantly reduced fasting blood glucose levels and slightly decreased AUC-OGTT with no remarkable difference relative to the ob/ob group (Figures 4B,C). The mice were subjected to ITTs on the 14th week of drug treatment to assess insulin tolerance. We observed that ob/ob mice showed insulin intolerance, and the AUC-ITT was significantly higher than that of lean WT mice, which was obviously mitigated in the RMA or MLF group. Similarly, RMA, MLF and MLP treatment significantly decreased serum insulin levels in ob/ob mice (Figures 4F,G). Subsequently, insulin sensitivity was assessed by HOMA-IR and ISI. As expected, treatment with RMA, MLF and MLP in ob/ob mice significantly reduced HOMA-IR and enhanced HOMA-ISI (Figures 4H,I) indicating that RMA, MLF and MLP elevate insulin sensitivity in ob/ob mice. Taken together, these results suggested that RMA and MLF treatment both ameliorate glucose and insulin intolerance in ob/ob mice, whereas MLF has a more significant effect.

### MLE and MLP ameliorate hepatic lipid accumulation in ob/ob mice

To evaluate the effects of RMA, MLF, and MLP on ob/ob mouse hepatic lipid accumulation, we stained the liver tissues with H&E. We observed that the livers of lean WT mice showed an utterly normal structure with distinguishable edges and a clear outline. However, the livers of ob/ob mice had severe hepatic lipid accumulation, the hepatic lobular structure was unclear, and the cytoplasm was filled with a large number of fat vacuoles. RMA treatment did not influence the accumulation of intrahepatic lipids in ob/ob mice, while MLF and MLP treatments reduced fat vacuoles and ameliorated hepatic lipid accumulation (Figure 4J).

### Screened compounds of MLF and RMA and T2DM targets

**FIGURE 5:**
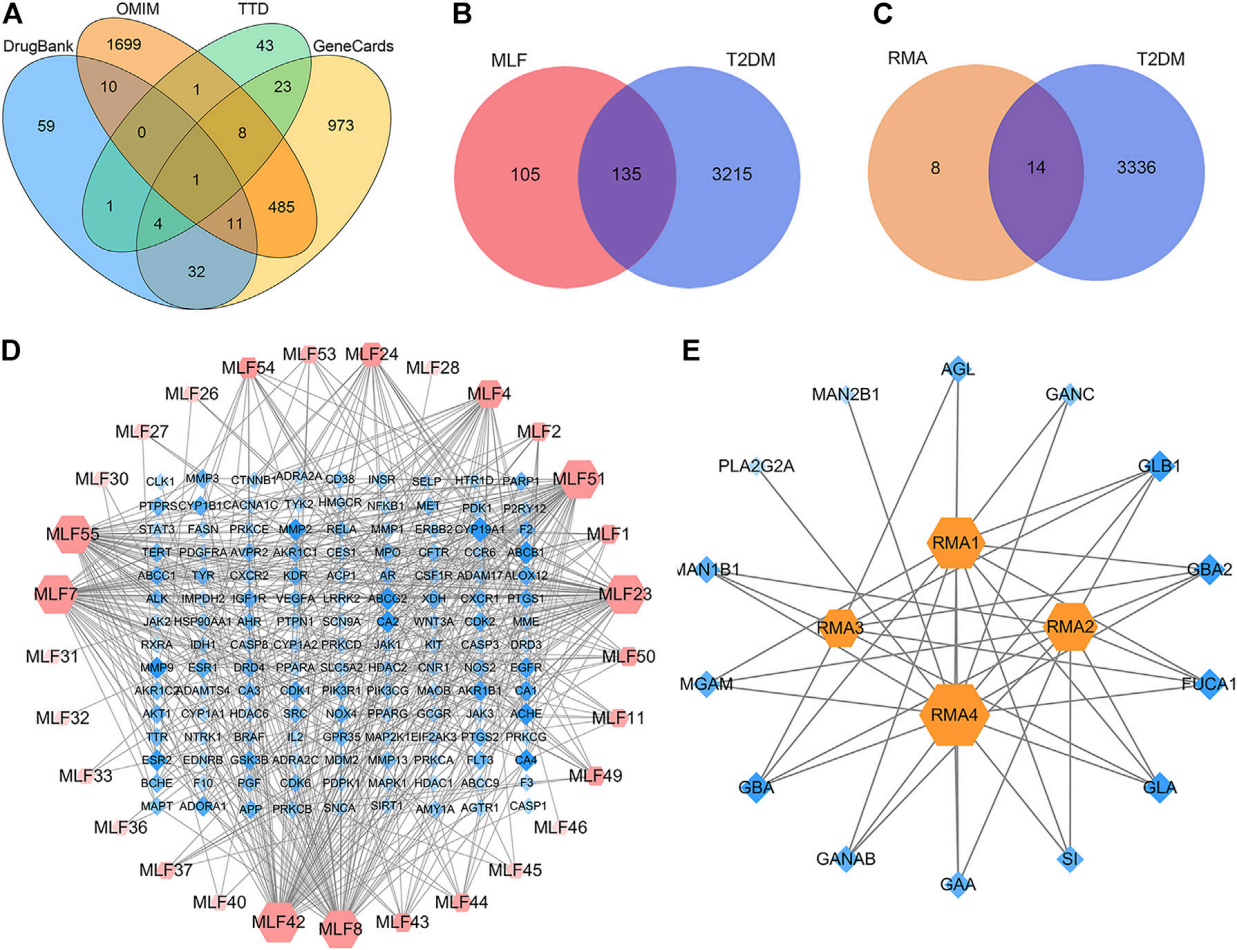
Target or active compound prediction and network construction. **(A)** Putative targets for T2DM were retrieved from the Drugbank, OMIM, TTD and GeneCards databases. **(B)** The 135 matched targets common between the predicted mulberry leaf flavonoid targets and the type 2 diabetes targets. **(C)** The 14 matched targets common between the predicted Ramulus Mori alkaloid targets and the type 2 diabetes targets. **(D)** The compound-target network implicated in type 2 diabetes using mulberry leaf flavonoids. The red nodes represent the active mulberry leaf flavonoids, whereas the blue nodes represent the antidiabetic targets of the active compounds. **(E)** The compound-target network implicated in type 2 diabetes using the *Ramulus Mori* alkaloids. The orange nodes represent the active *Ramulus Mori* alkaloids, whereas the blue nodes represent the antidiabetic targets of the active compounds. The edges represent the interactions between compounds and targets, and the node size is proportional to the degree of interaction.

Network pharmacology was conducted to investigate the mechanism of MLF and RMA in the treatment of T2DM. A total of 29 flavonoids of *Morus alba* L. were collected from the TCMSP database and published literature (Yang, 2010; Chen, 2014; Li, 2017) (Table 2). SwissTargetPrediction was used for target prediction, and the results with a probability >0.1 were selected for subsequent analysis. With repeat targets excluded, 240 drug targets were ultimately obtained. We retrieved a total of 3,350 putative targets of T2DM from the DrugBank, OMIM, TTD and GeneCards databases (Figure 5A). We compared these targets with the predicted MLF targets, and 135 common targets were filtered as the key targets for testing the antidiabetic activity of the MLF (Figure 5B). Subsequently, we constructed a compound-protein network based on the 135 overlapped targets and their corresponding compounds, composed of 164 nodes and 489 edges (Figure 5D). Network analysis revealed that the average degree of the 29 flavonoids was 16.8. We obtained 8 compounds (morusin, kaempferol, quercetin, norartocarpetin, kuwanon C, morusyunnansin L, morin, and fisetin) that had degree values higher than the average degree of 16.8. Therefore, these 8 compounds were regarded as potential bioactive compounds of MLF against T2DM. Moreover, we obtained 4 alkaloids of *Ramulus Mori* (Table 2) from published literature (Chen et al., 2000; Yang et al., 2015) and a total of 22 targets were predicted through a chemical similarity-based target search. We constructed a Venn diagram based on the targets of RMA and T2DM and obtained 14 overlapping targets that were considered the potential targets of RMA against diabetes (Figure 5C). A compound-target network comprising 18 nodes and 42 edges was then constructed (Figure 5E). Network analysis showed that DNJ (RMA1, degree = 11) and N-methyl-1-deoxynojirimycin (RMA4, degree = 14) had the highest numbers of connections to different targets. Taken together, we screened 135 targets and 8 compounds in MLF and 14 targets and 2 compounds in RMA by network pharmacology analysis.

**TABLE 2:** The 29 active compounds of mulberry (*Morus alba* L.) leaf flavonoids and 4 active compounds of *Ramulus Mori* alkaloids.

ID	Name	2D structure	Source
MLF1	2-(8-(2-hydroxypropan-2-yl)-3,4,8,9-tetrahydro-2H-furo[2,3-h]chromen-2-yl)-5-methoxyphenol	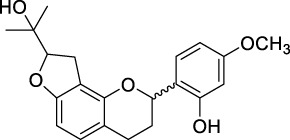	published literature
MLF2	2-(2-hydroxy-4-methoxyphenyl)-8,8-dimethyl-3,4,9,10-tetrahydro-2H,8H-pyrano[2,3-f]chromen-9-ol	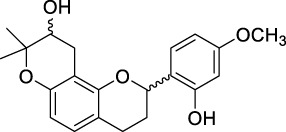	published literature
MLF4	Morusin	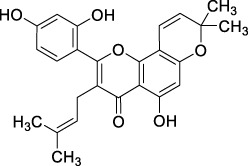	published literature
MLF7	Kaempferol	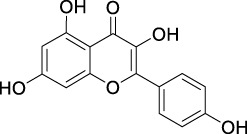	TCMSP
MLF8	Quercetin	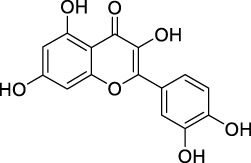	TCMSP
MLF11	Rutin	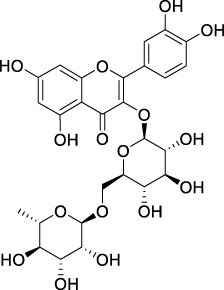	published literature
MLF23	Norartocarpetin	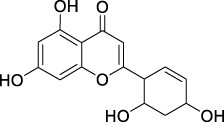	TCMSP
MLF24	Kuwanon C	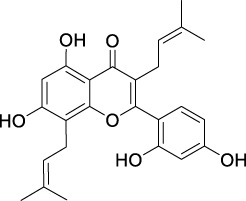	published literature
MLF26	Mornigrol F	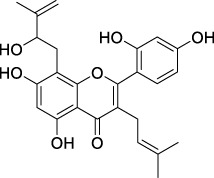	published literature
MLF27	Mornigrol G	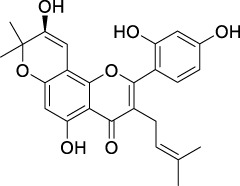	published literature
MLF28	6-geranylapigenin	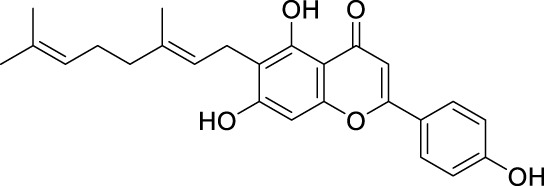	published literature
MLF30	(2S)-2′,4-dihydroxy-7-methoxy-8-yl butyrate flavan	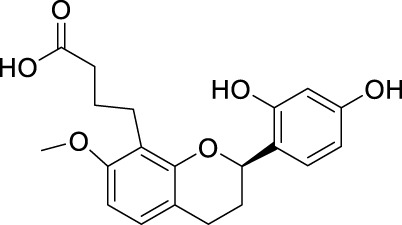	published literature
MLF31	isopentenyl-2′,4′-dihydroxy-7-methoxy flavan	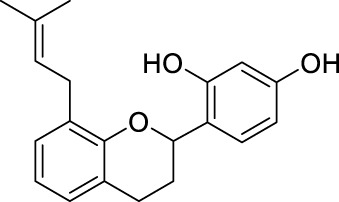	published literature
MLF32	isopentenyl-7,2c--dihydroxy-4′-methoxy flavan	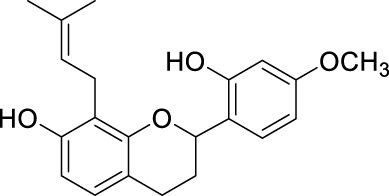	published literature
MLF33	Brosimine B	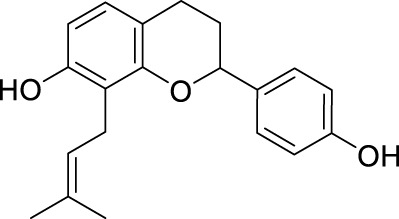	published literature
MLF36	Morachalcone A	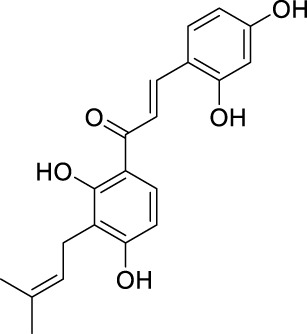	published literature
MLF37	Isobavaehaleone	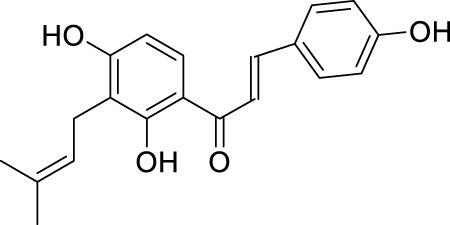	published literature
MLF40	Morusyunnansin J	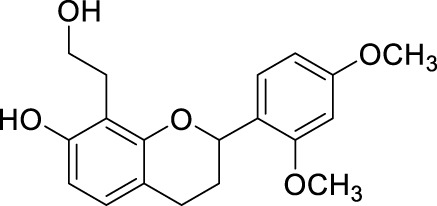	published literature
MLF42	Morusyunnansin L	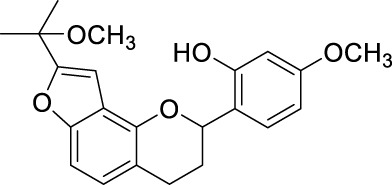	published literature
MLF43	Morusyunnansin M	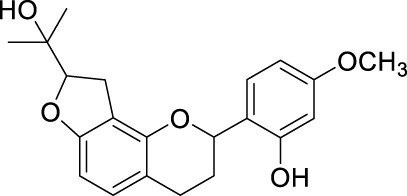	published literature
MLF44	Morusyunnansin N	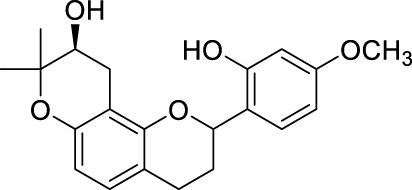	published literature
MLF45	(2S)-7,2′-dihydroxy-4′-methoxy-8-prenylflavan	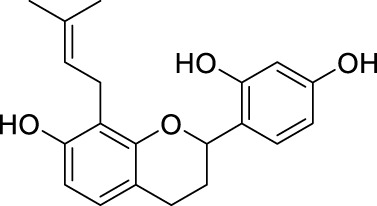	published literature
MLF46	(2S)-2′,4′-dihydroxy-7-methoxy-8-prenylflavan	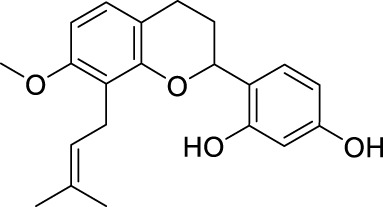	published literature
MLF49	2,4,2′,4′-tetrahydroxychalcone	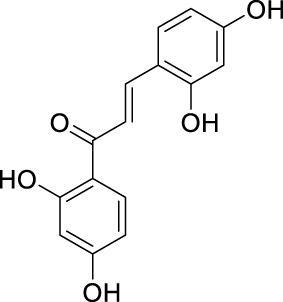	published literature
MLF50	Euchrenone a7	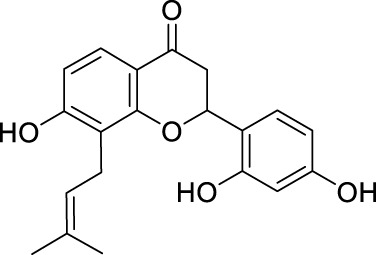	published literature
MLF51	Morin	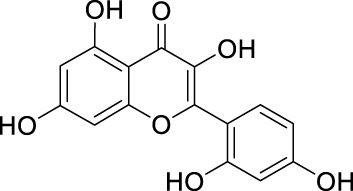	published literature
MLF53	Iristectorigenin A	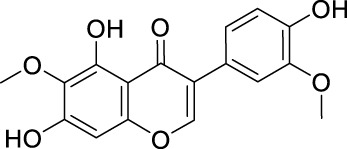	published literature
MLF54	Tetramethoxyluteolin	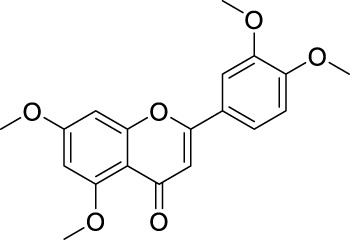	published literature
MLF55	Fisten	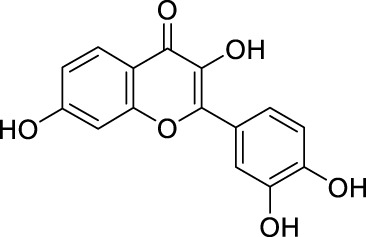	published literature
RMA1	1-deoxynojirimycin	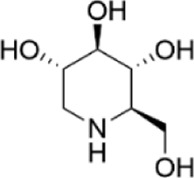	published literature
RMA2	Fagomine	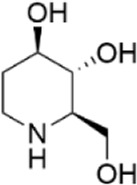	published literature
RMA3	1,4-dideoxy-1,4-imino-D-arabinitol	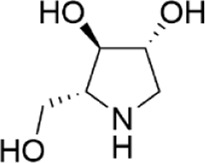	published literature
RMA4	N-methyl-1-deoxynojirimycin	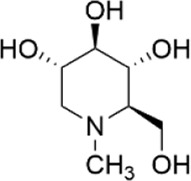	published literature

### PPI network of the anti-diabetic targets of MLF and RMA

**FIGURE 6:**
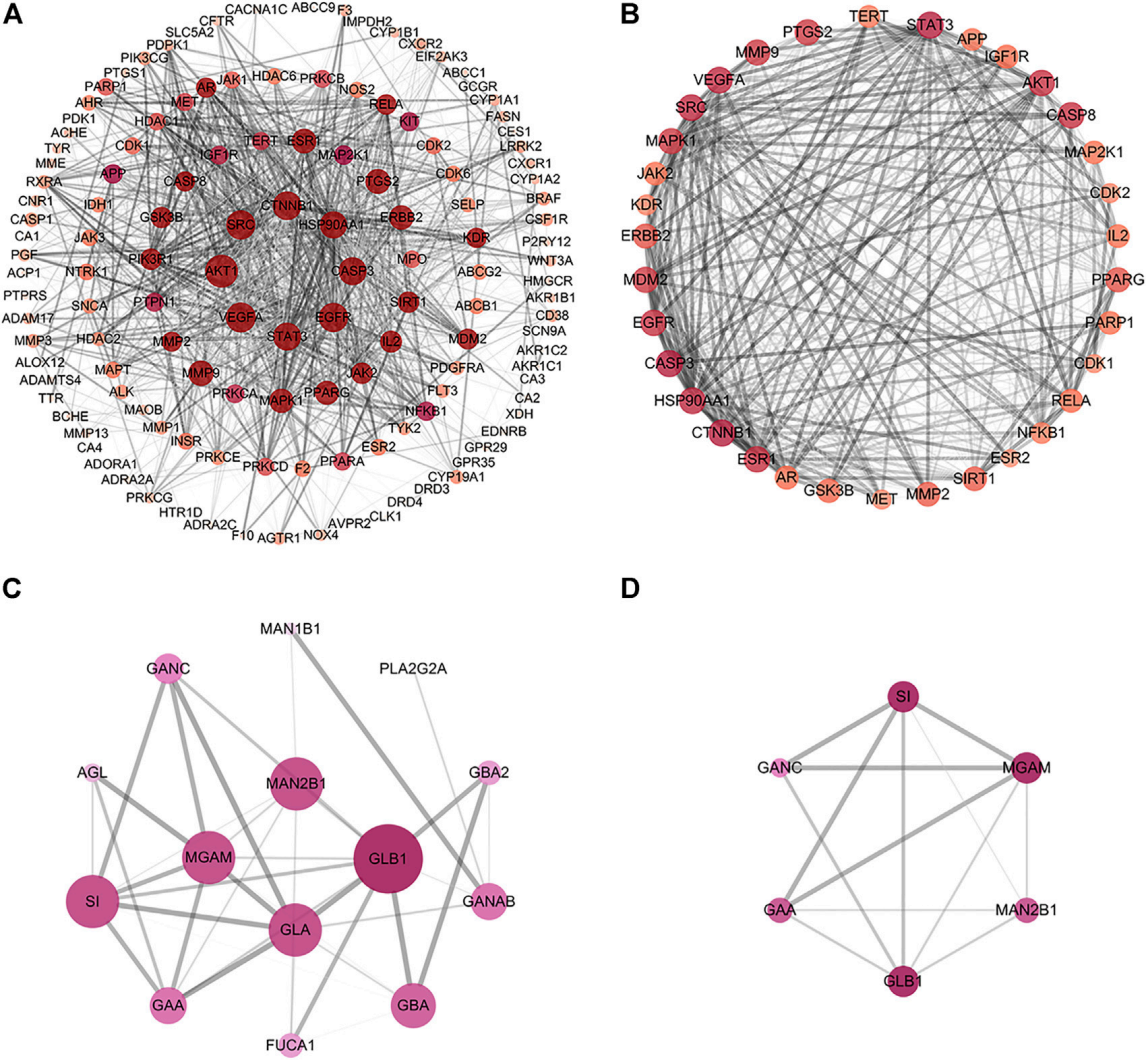
Construction of the PPI network and core targets. **(A)** The PPI network of potential protein targets of MLF in the treatment of T2DM constructed using Cytoscape and analyzed using NetworkAnalyzer. **(B)** MCODE cluster generated from **(A)**. **(C)** PPI network of potential targets of RMA in the treatment of T2DM. **(D)** MCODE cluster generated from **(C)**. The depth of color represents the degree value, and the node size is proportional to the degree of interaction.

To explore the PPI relationships of 135 potential protein targets of MLF and 14 targets of RMA related to the treatment of T2DM, we imported these data into the STRING database for analysis. Then, the .tsv file of the PPI data generated in STRING was input into Cytoscape (version 3.7.1) to construct a more intuitive network (Figures 6A,C). In the PPI network, a node with a larger size and deeper color possesses a higher degree value. The PPI network of MLF comprised 134 nodes (1 disconnected node was deleted) and 1,552 edges (Figure 6A), whereas the PPI network of RMA involved 14 nodes and 40 edges (Figure 6C). Similar functional clusters of the PPI network were selected by MCODE analysis using Cytoscape 3.7.1 software, and the attribute values of the cluster are listed in Table 3. The MLF cluster contained 34 nodes and 446 edges (Figure 6B). The average values of degree centrality, betweenness centrality, and closeness centrality were 26.23529412, 0.006405972, and 0.842350158, respectively. We found 16 targets whose degree centrality, betweenness centrality, and closeness centrality values were greater than the average. The 16 targets were PTGS2, SRC, MDM2, ESR1, AKT1, VEGFA, CASP8, MMP9, MAPK1, PPARG, STAT3, ERBB2, EGFR, CASP3, HSP90AA1 and CTNNB1 (Figure 6B). Moreover, for RMA, the cluster consisted of 6 nodes and 13 edges, and the average values of degree centrality, betweenness centrality, and closeness centrality were 4.333333, 0.033333335, and 0.896825395, respectively. The top 3 hub targets of RMA were MGAM, GLB1 and SI (Figure 6D). It is believed that these hub targets play a major role in treating T2DM by MLF and RMA.

**TABLE 3:** The cluster network parameters of mulberry (*Morus alba* L.) leaf flavonoids and *Ramulus Mori* alkaloids.

Network parameters	Value
Number of nodes	34
Number of edges	446
Clustering coefficient	0.850
Network diameter	2
Network radius	1
Network centralization	0.218
Network density	0.795
Shortest paths	1,122 (100%)
Characteristic path length	1.205
Avg. number of neighbors	26.235
Number of nodes	6
Number of edges	13
Clustering coefficient	0.9
Network diameter	2
Network radius	1
Network centralization	0.200
Network density	0.867
Shortest paths	30 (100%)
Characteristic path length	1.133
Avg. number of neighbors	4.333

### GO enrichment analysis

**FIGURE 7:**
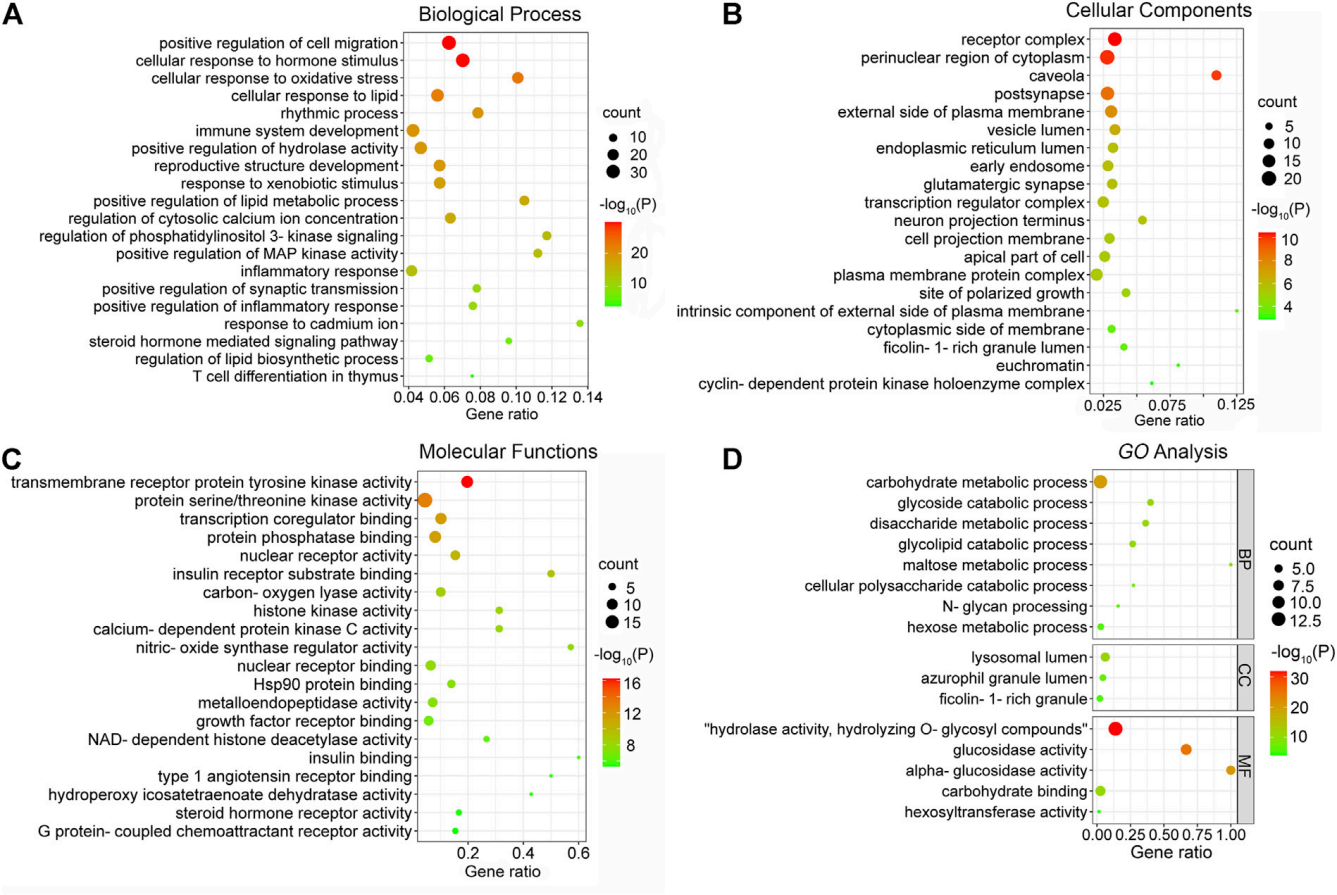
GO enrichment analysis of the antidiabetic targets of MLF and RMA. **(A)** Biological processes of MLF; **(B)** cellular components of MLF; **(C)** molecular function of MLF; **(D)** GO analysis of RMA.

To further explore the mechanisms of MLF and RMA in T2DM, we performed a GO enrichment analysis of the 135 predicted targets of MLF and the 14 potential targets of RMA. Our results revealed the top 20 enriched GO terms of biological process (BP), cellular component (CC), and molecular function (MF) of MLF and RMA (Figure 7). According to the BP results (Figure 7A), the functions of active compounds of MLF in T2DM mainly focused on cell migration, lipid metabolic process and hydrolase activity, response to hormone stimulus, oxidative stress and lipid, and were involved in rhythmic processes and immune system development. The CC results mainly included receptor complex, perinuclear region of cytoplasm, caveola, postsynapse, and the external side of the plasma membrane (Figure 7B). For MF (Figure 7C), the targets mostly involved transmembrane receptor protein tyrosine kinase activity, protein serine/threonine kinase activity, transcription coregulator binding, nuclear receptor activity and insulin receptor substrate binding. The analyses above showed that these targets are closely related to the processes of regulating kinase activity, lipid metabolism, and the insulin signaling pathway. The top three BP terms of RMA were carbohydrate metabolic process, glycoside catabolic process and glycolipid catabolic process (Figure 7D). The CC results included lysosomal lumen, azurophil granule lumen and ficolin-1-rich granule (Figure 7D). For MF, the top 3 terms were hydrolase activity, hydrolyzing O-glycosyl compounds, glucosidase activity and alpha-glucosidase activity (Figure 7D). These results suggested that the potential targets of RMA are highly associated with carbohydrate metabolism and the regulation of alpha-glucosidase activity.

### KEGG enrichment analysis

**FIGURE 8:**
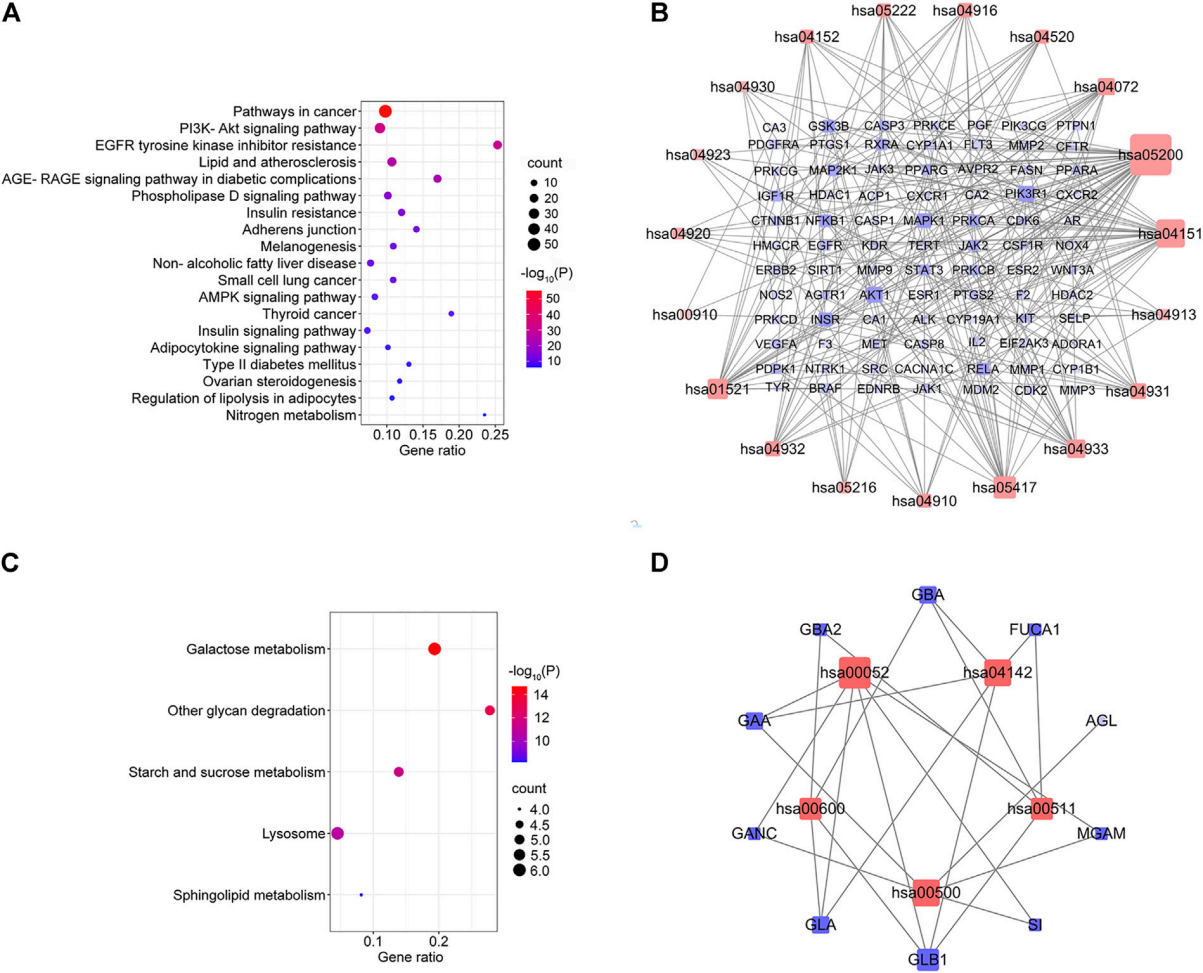
KEGG pathway enrichment analysis of the anti-diabetes targets of MLF and RMA. **(A)** Pathway enrichment results of MLF at *p* < 0.01. **(B)** The target–pathway network implicated in the mechanism of MLF in type 2 diabetes treatment. **(C)** Pathway enrichment results of RMA at *p* < 0.01. **(D)** The target–pathway network implicated in the mechanism of RMA in type 2 diabetes treatment. The red nodes represent the pathways, whereas the blue nodes represent the targets involved in these pathways. The edges represent the interactions between the targets and the pathways, and the node size is proportional to the degree of interaction.

KEGG pathway enrichment analysis was applied to explore the functions and signaling pathways of MLF and RMA antidiabetic targets. The top 20 KEGG pathways of MLF targets include the PI3K- Akt signaling pathway (hsa04151), lipid and atherosclerosis (hsa05417), the AGE-RAGE signaling pathway in diabetic complications (hsa04933), insulin resistance (hsa04931), and type 2 diabetes mellitus (hsa04930). Consistent with the above GO analysis, they were closely related to glucose and lipid metabolism, insulin signaling and oxidative stress (Figure 8A). Then, based on the number of targets involved in each pathway, a target-pathway network was constructed using Cytoscape (version 3.7.1) (Figure 8B). Most targets were mainly enriched in pathways in cancer, PI3K- Akt signaling, and lipid and atherosclerosis. In addition, AKT1, MAPK1, PIK3R1 and INSR participated in the greatest numbers of pathways. The top three significant KEGG pathways of RMA were galactose metabolism (hsa00052), other glycan degradation (hsa00511), and starch and sucrose metabolism (hsa00500) (Figure 8C; Table 4). The target-pathway network of RMA showed that GLB1, GLA, GBA and GAA are the core targets involved in the majority of pathways (Figure 8D). Combined with the GO analysis results, these results suggest that the mechanism of action of RMA improves insulin resistance and glucose tolerance through influencing carbohydrate metabolism by regulating alpha-glucosidase activity.

**TABLE 4:** Annotation of KEGG pathways of mulberry (*Morus alba* L.) leaf flavonoids and *Ramulus Mori* alkaloids.

Term ID	Description	Count	*p* Value	Genes
hsa05200	Pathways in cancer	52	9.87066E-56	AGTR1, AKT1, ALK, AR, BRAF, CASP3, CASP8, CDK2, CDK6, CSF1R, CTNNB1, EDNRB, EGFR, ERBB2, ESR1, ESR2, F2, FLT3, GSK3B, HDAC1, HDAC2, HSP90AA1, IGF1R, IL2, JAK1, JAK2, JAK3, KIT, MDM2, MET, MMP1, MMP2, MMP9, NFKB1, NOS2, NTRK1, PDGFRA, PGF, PIK3R1, PPARG, PRKCA, PRKCB, PRKCG, MAPK1, MAP2K1, PTGS2, RELA, RXRA, STAT3, TERT, VEGFA, WNT3A
hsa04920	Adipocytokine signaling pathway	7	2.6053E-08	AKT1, JAK2, NFKB1, PPARA, RELA, RXRA, STAT3
hsa01521	EGFR tyrosine kinase inhibitor resistance	20	4.93654E-30	AKT1, BRAF, EGFR, ERBB2, GSK3B, IGF1R, JAK1, JAK2, KDR, MET, PDGFRA, PIK3R1, PRKCA, PRKCB, PRKCG, MAPK1, MAP2K1, SRC, STAT3, VEGFA
hsa04932	Non-alcoholic fatty liver disease	12	5.81113E-12	AKT1, CASP3, CASP8, GSK3B, INSR, NFKB1, PIK3R1, PPARA, PPARG, RELA, RXRA, EIF2AK3
hsa05216	Thyroid cancer	7	2.7995E-10	BRAF, CTNNB1, NTRK1, PPARG, MAPK1, MAP2K1, RXRA
hsa04910	Insulin signaling pathway	10	6.41988E-10	AKT1, BRAF, FASN, GSK3B, INSR, PDPK1, PIK3R1, MAPK1, MAP2K1, PTPN1
hsa05417	Lipid and atherosclerosis	23	3.16371E-25	AKT1, CASP1, CASP3, CASP8, CYP1A1, GSK3B, HSP90AA1, JAK2, MMP1, MMP3, MMP9, NFKB1, PDPK1, PIK3R1, PPARG, PRKCA, MAPK1, RELA, RXRA, SELP, SRC, STAT3, EIF2AK3
hsa04933	AGE-RAGE signaling pathway in diabetic complications	17	1.93973E-22	AGTR1, AKT1, CASP3, F3, JAK2, MMP2, NFKB1, PIK3R1, PRKCA, PRKCB, PRKCD, PRKCE, MAPK1, RELA, STAT3, VEGFA, NOX4
hsa04931	Insulin resistance	13	2.25338E-15	AKT1, GSK3B, INSR, NFKB1, PDPK1, PIK3R1, PPARA, PRKCB, PRKCD, PRKCE, PTPN1, RELA, STAT3
hsa04520	Adherens junction	10	8.29E-13	ACP1, CTNNB1, EGFR, ERBB2, IGF1R, INSR, MET, MAPK1, PTPN1, SRC
hsa04913	Ovarian steroidogenesis	6	1.08695E-07	CYP1A1, CYP1B1, CYP19A1, IGF1R, INSR, PTGS2
hsa04151	PI3K-Akt signaling pathway	32	1.3511E-32	AKT1, CDK2, CDK6, CSF1R, EGFR, ERBB2, FLT3, GSK3B, HSP90AA1, IGF1R, IL2, INSR, JAK1, JAK2, JAK3, KDR, KIT, MDM2, MET, NFKB1, NTRK1, PDGFRA, PDPK1, PGF, PIK3CG, PIK3R1, PRKCA, MAPK1, MAP2K1, RELA, RXRA, VEGFA
hsa04072	Phospholipase D signaling pathway	15	2.02471E-16	AGTR1, AKT1, AVPR2, EGFR, F2, CXCR1, CXCR2, INSR, KIT, PDGFRA, PIK3CG, PIK3R1, PRKCA, MAPK1, MAP2K1
hsa04916	Melanogenesis	11	1.05866E-12	CTNNB1, EDNRB, GSK3B, KIT, PRKCA, PRKCB, PRKCG, MAPK1, MAP2K1, TYR, WNT3A
hsa05222	Small cell lung cancer	10	1.19614E-11	AKT1, CASP3, CDK2, CDK6, NFKB1, NOS2, PIK3R1, PTGS2, RELA, RXRA
hsa04152	AMPK signaling pathway	10	1.73256E-10	AKT1, CFTR, FASN, HMGCR, IGF1R, INSR, PDPK1, PIK3R1, PPARG, SIRT1
hsa04930	Type II diabetes mellitus	6	5.75767E-08	CACNA1C, INSR, PIK3R1, PRKCD, PRKCE, MAPK1
hsa04923	Regulation of lipolysis in adipocytes	6	1.92408E-07	ADORA1, AKT1, INSR, PIK3R1, PTGS1, PTGS2
hsa00910	Nitrogen metabolism	4	8.67479E-07	CA1, CA2, CA3, CA4
hsa00052	Galactose metabolism	6	2.08301E-15	GAA, GANC, GLA, GLB1, SI, MGAM
hsa00500	Starch and sucrose metabolism	5	3.57233E-12	AGL, GAA, GANC, SI, MGAM
hsa00511	Other glycan degradation	5	8.15532E-14	FUCA1, GBA, GLB1, MAN2B1, GBA2
hsa04142	Lysosome	6	1.81021E-11	FUCA1, GAA, GBA, GLA, GLB1, MAN2B1
hsa00600	Sphingolipid metabolism	4	6.03913E-09	GBA, GLA, GLB1, GBA2

### Molecular docking findings

**FIGURE 9:**
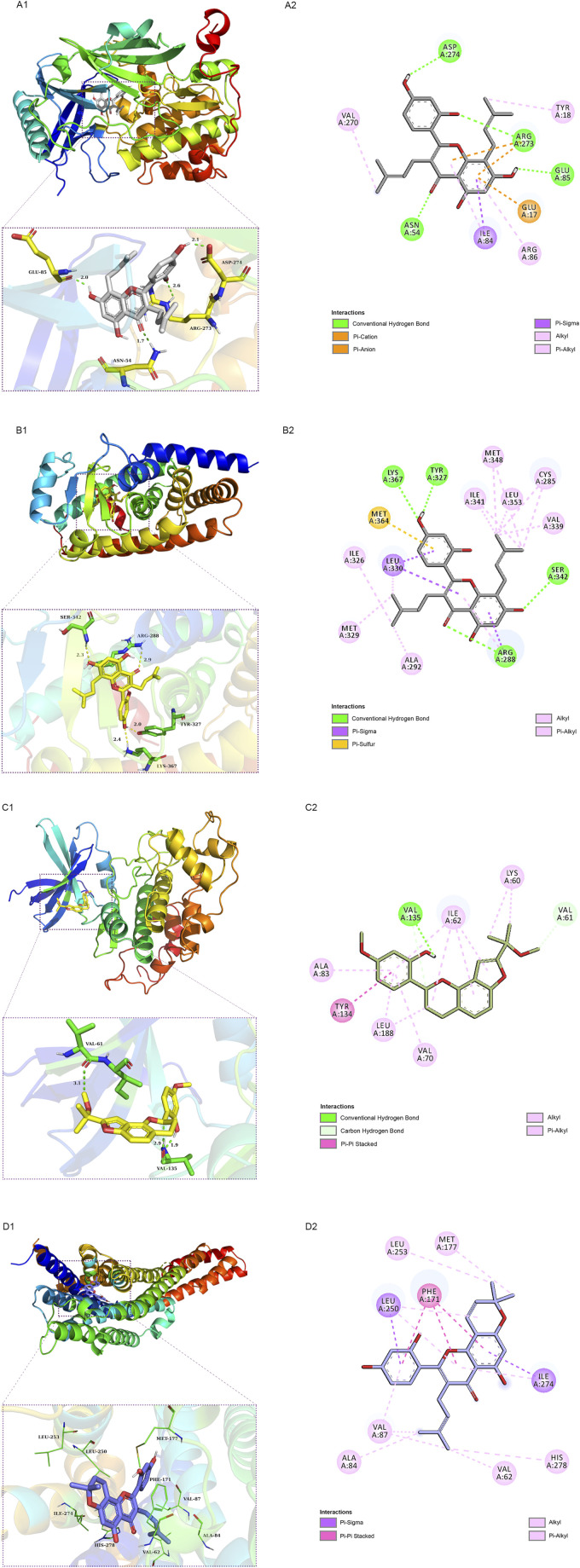
Schematic 2D and 3D representations of the molecular docking model and active sites. Binding modes of kuwanon C (MLF24) to AKT1 **(A1)**, kuwanon C (MLF24) to PPARγ **(B1)**, morusyunnansin L (MLF42) to GSK3β **(C1)**, and morusin (MLF4) to ADORA1**(D1)**. **(A2, B2, C2, D2)**: Two-dimensional patterns of bonds.

We employed molecular docking to analyze the possibility of binding between the core targets and the active compounds *via* AutoDockTools. A previous study proved that a binding affinity < −7.0 kcal/mol indicated that the two molecules had strong binding activity (Trott and Olson, 2010). In the current study, we docked two top targets in the insulin signaling pathway (serine/threonine-protein kinase, AKT and glycogen synthase kinase-3 beta, GSK3β), PPARγ and ADORA1 with active compounds of MLF. The results illustrated that most of the binding energies were < −7 kcal/mol, and the binding energies between PPARγ and MLF24 as well as ADORA1 and MLF4 were < −8.9 kcal/mol (Table 5). Therefore, diabetes-associated targets (AKT1 and PPARγ) and two targets involved in regulating glucolipid metabolism (GSK3β and ADORA1), which have the lowest free energy binding with their compounds, were selected for molecular docking and refined by exploring the specific binding sites. A lower free binding energy value indicates stronger binding to the target protein. The free binding energies of MLF24 with AKT1 and PPARγ were −8.51 and −8.99 kcal/mol, respectively. In AKT1, MLF24 had hydrogen bonding with the ASP-274, ARG-273, GLU-85 and ASN-54 residues; a Pi-anion interaction with the GLU-17 residue; a Pi-Sigma interaction with the ILT-84 residue; and hydrophobic interactions with the VAL-270, TYR-18 and ARG-86 residues of AKT1(Figures 9A1,A2). In PPARγ, the binding affinity was contributed by the following: hydrogen bonding with the LYS-367, TYR-327, SER-342 and ARG-288 residues; a Pi-Sigma interaction with the LEU-330 residue; a Pi-Sulfur interaction with the MET-364 residue; and hydrophobic interactions with the ILE-326, MET-329, ALA-292, ILE-341, MET-348, LEU-353, CYS-285 and VAL-339 residues of PPARγ (Figures 9B1,B2). MLF42 showed −8.44 kcal/mol with GSK3β and formed hydrogen binding, carbon‒hydrogen binding, and Pi-Pi stacking with residues VAL-135, VAL-61, and TYR-134, respectively. Through hydrophobic interactions MLF42 interacts with the ALA-83, LEU-188, VAL-70, ILE-62 and LYS-60 residues of GSK3β. MLF4 has solid binding interactions with ADORA1 (binding energy = -9.31 kcal/mol). MLF4 docked with six residues to form hydrophobic interactions in ADORA1 (LEU-253, MET-177, ALA-84, VAL-87, VAL-62 and HIS-278) and two Pi-Sigma interactions with the LEU-250 and ILE-274 residues, as well as Pi-Pi stacking interactions with the PHE-171 residue (Figures 9D1,D2). The molecular docking results suggested that hydrogen bonding and hydrophobic interactions were the main forms of interaction. Collectively, these results implied that kuwanon C, morusin and morusyunnansin L are the main compounds of MLF, which exert antidiabetic effects by regulating ATK1, PPARγ, ADORA1, and GSK3β, respectively.

**TABLE 5:** Free binding energies of AKT1, PPARG, GSK3β and ADORA1 with their corresponding active compounds.

Target	Compound	Free binding energy (kcal/mol)
AKT1	MLF24	−8.51
	MLF42	−8.05
	MLF8	−7.59
	MLF55	−7.47
	MLF7	−7.37
	MLF23	−6.89
PPARG	MLF24	−8.99
GSK3β	MLF42	−8.44
	MLF55	−7.71
	MLF8	−7.6
	MLF7	−7.37
	MLF23	−7.36
	MLF51	−7.36
	MLF54	−7.12
ADORA1	MLF4	−9.31
	MLF24	−8.12
	MLF54	−7.53
	MLF55	−7.25
	MLF8	−6.89
	MLF51	−6.80
	MLF23	−6.72
	MLF7	−6.37

### Validation of the key targets in ob/ob mice

**FIGURE 10:**
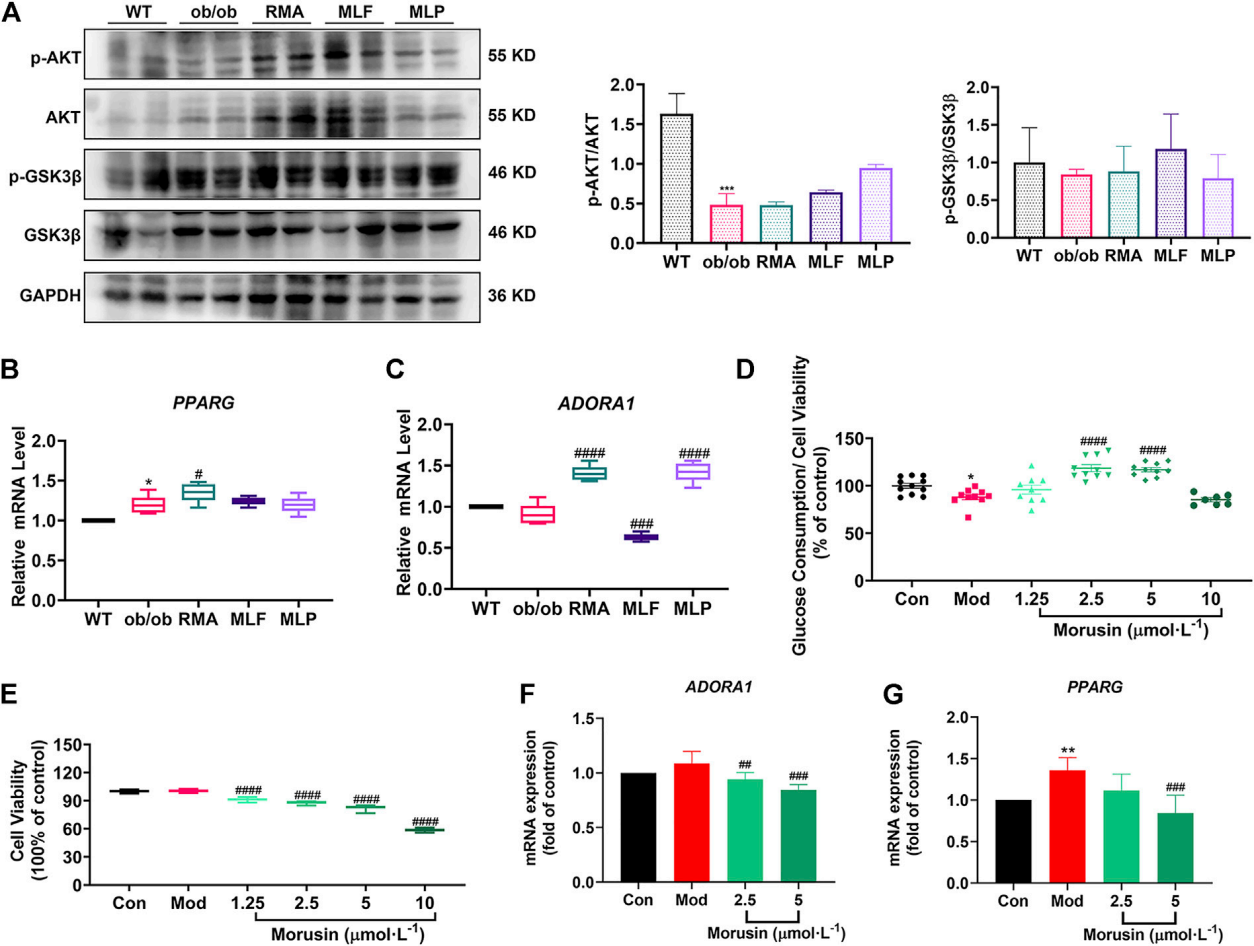
Validation of key targets in ob/ob mice and insulin-resistant L02 cells. **(A)** Protein levels of p-AKT, AKT, p-GSK3β and GSK3β in the livers of ob/ob mice. mRNA levels of *PPARG* **(B)** and *ADORA1* **(C)** in the livers of ob/ob mice. *p < 0.05, ***p < 0.001, compared with WT group; ^#^ *p* < 0.05, ^###^ *p* < 0.001, ^####^ *p* < 0.0001, compared with the ob/ob group. Glucose consumption **(D)** and cell viability **(E)** were determined following treatment with different concentrations of morusin in L02 cells. RT‒qPCR analysis of mRNA levels of *ADORA1* **(F)** and *PPARG* **(G)** in insulin‒resistant L02 cells stimulated with morusin for 24 h *p < 0.05, **p < 0.01, compared with Con group; ^##^ *p* < 0.01, ^###^ *p* < 0.001, ^####^ *p* < 0.0001, compared with Mod group.

Validation of the key targets was carried out in ob/ob mice. Compared with WT mice, the phosphorylated protein levels of AKT (Ser473) and GSK3β (Ser9) were reduced in the livers of ob/ob mice (Figure 10A). However, MLF treatment increased the protein expression levels of p-AKT and p-GSK3β in ob/ob mice (Figure 10A). In addition, the protein expression levels of p-AKT and p-GSK3β were not obviously influenced by RMA treatment (Figure 10A). MLP treatment increased the phosphorylation of AKT, but had no apparent effect on the protein expression levels of p-GSK3β (Figure 10A). In addition, compared with WT mice, the transcriptional level of *PPARG* in the livers of ob/ob mice was significantly increased (Figure 10B). Surprisingly, RMA treatment increased the expression level of *PPARG*, while MLF and MLP treatment had no effect on the expression of the *PPARG* gene (Figure 10B). Moreover, there was no apparent difference in the gene expression level of *ADORA1* between ob/ob mice and WT mice (Figure 10C). However, RMA and MLP treatments significantly increased the expression level of the *ADORA1* gene, and MLF treatment markedly reduced the gene expression level of *ADORA1* in the livers of ob/ob mice (Figure 10C).

### Morusin facilitates glucose consumption and represses the gene expression of *ADORA1* and *PPARG* in L02 cells

Our results indicated that morusin (2.5 and 5 μmol/L) markedly reversed cellular insulin resistance and reliably facilitated glucose consumption (Figure 10D). In addition, morusin significantly decreased the cell survival rate in a concentration range (1.25, 2.5, 5 and 10 μmol/L) (Figure 10E). We next examined the potential effects of morusin on *ADORA1* and *PPARG* gene expression. The expression level of *ADORA1* was slightly increased in the Mod group compared with the Con group, while 2.5 and 5 μmol/L morusin treatment observably repressed the expression of *ADORA1* (Figure 10F). Similarly, 5 μmol/L morusin also markably inhibited *PPARG* expression induced by insulin resistance (Figure 10G).

## Discussion

Natural products, including herbal formulas and their extracts, have been used to treat human diseases through unique systems of theories and therapies for thousands of years, and have also been increasingly applied to treat T2DM (Xu et al., 2018). Mulberry leaves, a traditional Chinese medicine, have been reported to reduce cholesterol levels, enhance high-density lipoprotein cholesterol, and decrease serum triglyceride and low-density lipoprotein cholesterol levels in patients with mild dyslipidemia (Aramwit et al., 2011; Aramwit et al., 2013). The functional components of mulberry leaves, mainly flavonoids, alkaloids and polysaccharides, account for approximately 0.7%–1.3%, 0.09–0.28%, and 1.2%–3.1% of their total dry weight, respectively (Liao et al., 2008; Lin et al., 2008; Pu, 2016; Ju et al., 2018). Our present results demonstrated that the glucose-lowering efficacy of MLF is comparable to those of RMA and MLP, while the lipid-lowering ability of MLF is superior to that of RMA after 14 weeks of treatment in ob/ob mice, suggesting that MLF holds better potential in the treatment of diabetes. Therefore, it is essential to explore the bioactive components and mechanisms of MLF and RMA for treating diabetes. We employed network pharmacology and molecular docking techniques to uncover the active ingredients, their potential targets, and the signaling pathways of MLF and RMA for the treatment of diabetes.

Network pharmacology was conducted on the 4 main ingredients (DNJ, FA, 1,4-dideoxy-1, 4-iminod-D-arabinitol and N-methyl-1-deoxynojirimycin) of RMA to predict the potential targets. Previous studies revealed that RMA had a high affinity for the disaccharidase active site and could selectively inhibit it (Liu Z. et al., 2019). Consistently, our PPI results suggested that MGAM and SI might be the key antidiabetic targets of RMA. Similarly, our GO analysis and KEGG pathway analysis results showed that RMA targeted hydrolase, glucosidase and alpha-glucosidase activity, and was involved in galactose, starch and sucrose metabolism, and other glycan degradation. Therefore, our current study suggests that RMA might exert hypoglycemic effects by inhibiting MGAM and SI to reduce alpha-glucosidase activity.

In contrast, MLF acts through a different mechanism. In the present study, we screened 26 active components of MLF and 135 hub targets *via* network pharmacology. The MLF compound–target network analysis indicated that morusin, kaempferol, quercetin, norartocarpetin, kuwanon C, morusyunnansin L, morin, and fisetin are the main antidiabetic active compounds of MLF. The PPI analysis shows that AKT1 and PPARγ are the targets with higher degrees in the cluster network. The serine/threonine kinase Akt, also known as protein kinase B, is a downstream effector of PI3K. Activated AKT modulates downstream targets and is involved in energy metabolism in the liver, skeletal muscle, and adipose tissue (Mora et al., 2004; Batista et al., 2021). PPARγ is highly expressed in adipose tissue and plays a vital role in maintaining glucose and lipid homeostasis by regulating genes involved in fatty acid transport and the triglyceride synthesis pathway (Lee et al., 2012). Glucose and lipid metabolic disorders are key to T2DM, while one of the main benefits of MLF is alleviating this disorder.

The GO enrichment analysis further revealed that MLF might regulate lipid metabolism, especially lipid biosynthetic processes, oxidative stress, inflammatory responses, and insulin signaling to improve metabolic disorders and exert antidiabetic effects. Abnormal lipid metabolism and inflammation are tightly associated with T2DM (Esser et al., 2014; Athyros et al., 2018). A previous study indicated that flavonoids from mulberry leaves could attenuate adiposity and regulate lipid metabolism in HFD-fed ICR mice (Zhong et al., 2020). Consistently, our present studies showed that MLF alleviates hepatic steatosis in ob/ob mice. Oxidative stress, induced by an abundance of reactive oxygen species or failure in the antioxidative machinery, has been considered a significant hallmark for the pathogenesis and development of T2DM (Rehman and Akash, 2017). Interestingly, numerous statistically significant BP terms, such as glucose metabolic process, glucose homeostasis, and response to glucose, were not at the top of the list. Taken together, we posit that the benefits of MLF in hypoglycemia are due not to lowering glucose profiles directly but to its effects on the insulin signaling pathway, lipid metabolism, and the inflammatory response.

To gain further insight into the mechanism, we obtained 20 signaling pathways and targets that MLF might correlate with the development of T2DM and its complications by KEGG pathway analysis. MLF impacted targets are widely involved in cancer, lipid metabolism, nonalcoholic fatty liver, insulin resistance and type 2 diabetes. For example, ADORA1 has been proven to be associated with lipid metabolism. ADORA1 is a G protein-coupled receptor family member and an important drug target for numerous diseases (Nieto Gutierrez and McDonald, 2018). Previous studies have shown that activation of ADORA1 in the central nervous system prevent**s** body weight gain by enhancing adipose sympathetic innervations to augment adipose tissue lipolysis (Zhang et al., 2021). In contrast, activation of ADORA1 in peripheral tissues could facilitate HFD-induced obesity in C57BL/6J mice (Zhang et al., 2021). Both mice fed the HFD diet and patients with hepatic steatosis showed increased hepatic ADORA1 expression. Specific inhibition of ADORA1 in the liver helps prevent body weight gain and alleviate hepatic steatosis (Hong et al., 2019). In the current study, we found that MLF treatment significantly reduced *ADORA1* mRNA expression in the livers of ob/ob mice. Therefore, we speculated that MLF might regulate lipid metabolism and alleviate hepatic steatosis by modulating the expression of ADORA1. To determine the interactions between compounds and their corresponding hub targets, we calculated their free binding energy and employed molecular docking to determine their binding mode. The results revealed that morusin (MLF4) displayed the highest affinity for the ADORA1 protein. Our verified experiments in human L02 hepatocytes revealed that the expression levels of *ADORA1* genes were upregulated in insulin resistance, while morusin treatment markedly increased cellular glucose consumption stimulated by insulin and downregulated *ADORA1* expression. Morusin is a prenylated flavone that allows for versatile salutary effects, including antioxidant, antitumor, and anti-inflammatory activities (Choi et al., 2020; Panek-Krzysko and Stompor-Goracy, 2021). Recently, the metabolically beneficial effects of morusin have been gradually recognized. Morusin downregulates the expression level of adipogenic transcription factors (PPARγ and C/EBPα) to inhibit lipid accumulation in 3T3-L1 adipocytes (Lee et al., 2018). MLF treatment had no obvious effect on *PPARG* gene expression in ob/ob mice. Notably, our results revealed that morusin showed higher binding activity with PPARγ, a regulator of adipocyte differentiation, lipid storage, glucose metabolism, and insulin sensitivity. Our *in vitro* results confirmed that morusin blocked the overexpression of *PPARG* caused by insulin resistance. Additionally, we found that kuwanon C (MLF24) also has a strong bond with PPARγ and ADORA1, suggesting that kuwanon C might be a potential selective modulator of PPARγ. A recent study demonstrated that mulberry leaves inhibited adipocyte differentiation and triglyceride synthesis by regulating the PPAR-γ-C/EBP-α (CCAAT/enhancer-binding protein type α) signaling pathway (Liao et al., 2021). Moreover, our current results revealed that morusin and kuwanon C might be important compounds of MLF that lower the levels of glucose and lipids, which play their role by targeting ADORA1 and PPARG.

Impaired insulin signaling is also central to the development of T2DM. Insulin binding to its receptor is the first step in activating the insulin signaling pathway, leading to tyrosine phosphorylation of IRS, which activates IRS and recruits PI3K to tyrosine-phosphorylated IRS (Saltiel and Kahn, 2001; Batista et al., 2021). Subsequently, phosphatidylinositol (3,4,5)-triphosphate (PIP3) was formed, and 3-phosphoinositide dependent protein kinase (PDK)-dependent AKT was activated, which then modulated the downstream targets and was involved in energy metabolism in liver, skeletal muscle and adipose tissue (Mora et al., 2004; Batista et al., 2021). In this study, treatment with MLF enhanced the phosphorylated protein levels of AKT (Ser473) and GSK3β (Ser9) in ob/ob mice. Moreover, we observed that kuwanon C also showed the highest affinity with AKT1, indicating that kuwanon C might target AKT1, regulate insulin signaling, and be involved in glucose metabolism. Notably, there has been no report about the bioactivity of morusyunnansin L. Our results revealed that morusyunnansin L exhibited a favorable molecular interaction with GSK3β. Therefore, we speculate that morusyunnansin L (MLF42) plays a hypoglycemic role by targeting GSK3β to modulate glycogen synthesis and increase glucose utilization.

## Conclusion

In the present study, we have experimentally demonstrated that MLF and MLP predominantly enhance glucose uptake in insulin-resistant human L02 hepatocytes. The glucose-lowering efficacies of MLF and MLP in ob/ob mice are comparable to that of RMA, while the lipid-lowering effects of MLF and MLP are superior to that of RMA, suggesting the potential of MLF and MLP in antidiabetes and antiobesity. A recent study has shown that MLF improves high-fat -diet-induced glycolipid metabolic abnormalities in mice by mediating gut microbiota, although the specific hypoglycemic active ingredients and targets of MLF remain unclear (Zhong et al., 2020). Here, our study revealed that DNJ, FA, and N-methyl-1-deoxynojirimycin are the primary active ingredients of RMA and target MGAM and SI proteins to lower glucose, whereas morusin, kuwanon C and morusyunnansin L are the key active compounds of MLF and play their hypoglycemic roles by targeting key proteins involved in lipid metabolism (ADORA1 and PPARγ) and insulin signaling (AKT1 and GSK3β). Additionally, we validated the hypoglycemic effects of morusin on repressing the expression of the *ADORA1* and *PPARG* genes to improve insulin resistance in L02 cells. Collectively, our results shed light on the mechanisms behind the glucose-lowering effects of MLF, suggesting that morusin and kuwanon C might be selective PPARγ modulators and possess broad prospects as new drugs or leads against diabetes.

## Data Availability

The original contribution presented in the study are included in the article/Supplementary Material, further inquiries can be directed to the corresponding authors.

## Abbreviations

ADORA1, Adenosine A1 receptor; AKT1, AKT serine/threonine kinase 1; DNJ, 1-deoxynojirimycin; FA, fagomine; GSK3β, glycogen synthase kinase 3 beta; HPLC, high-performance liquid chromatography; HOMA-IR, homeostasis model assessment-insulin resistance; HOMA-ISI, homeostasis model assessment-insulin sensitive index; ITT, insulin tolerance tests; LCA, lithocholic acid; MLE, the crude extract of mulberry leaf; MLF, mulberry leaf’s total flavonoids; MLP, mulberry leaf polysaccharides; MGAM, maltase-glucoamylase; OGTT, oral glucose tolerance tests; PPARγ, peroxisome proliferator-activated receptor gamma; PPI, protein-protein interaction; RMA, Ramulus Mori (Sangzhi) alkaloid; SI, sucrase-isomaltase; T2DM, type 2 diabetes mellitus.
