# Supplementary material for: Novel active compounds and the anti-diabetic mechanism of mulberry leaves
Source: Front Pharmacol. 2022 Oct 5;13:986931. doi: 10.3389/fphar.2022.986931 (PMC9581293; doi:10.3389/fphar.2022.986931)
Supplement: Supplementary file 1 [file DataSheet1.docx]

Supplementary Material

# Supplementary Figures and Tables

## Supplementary Figures


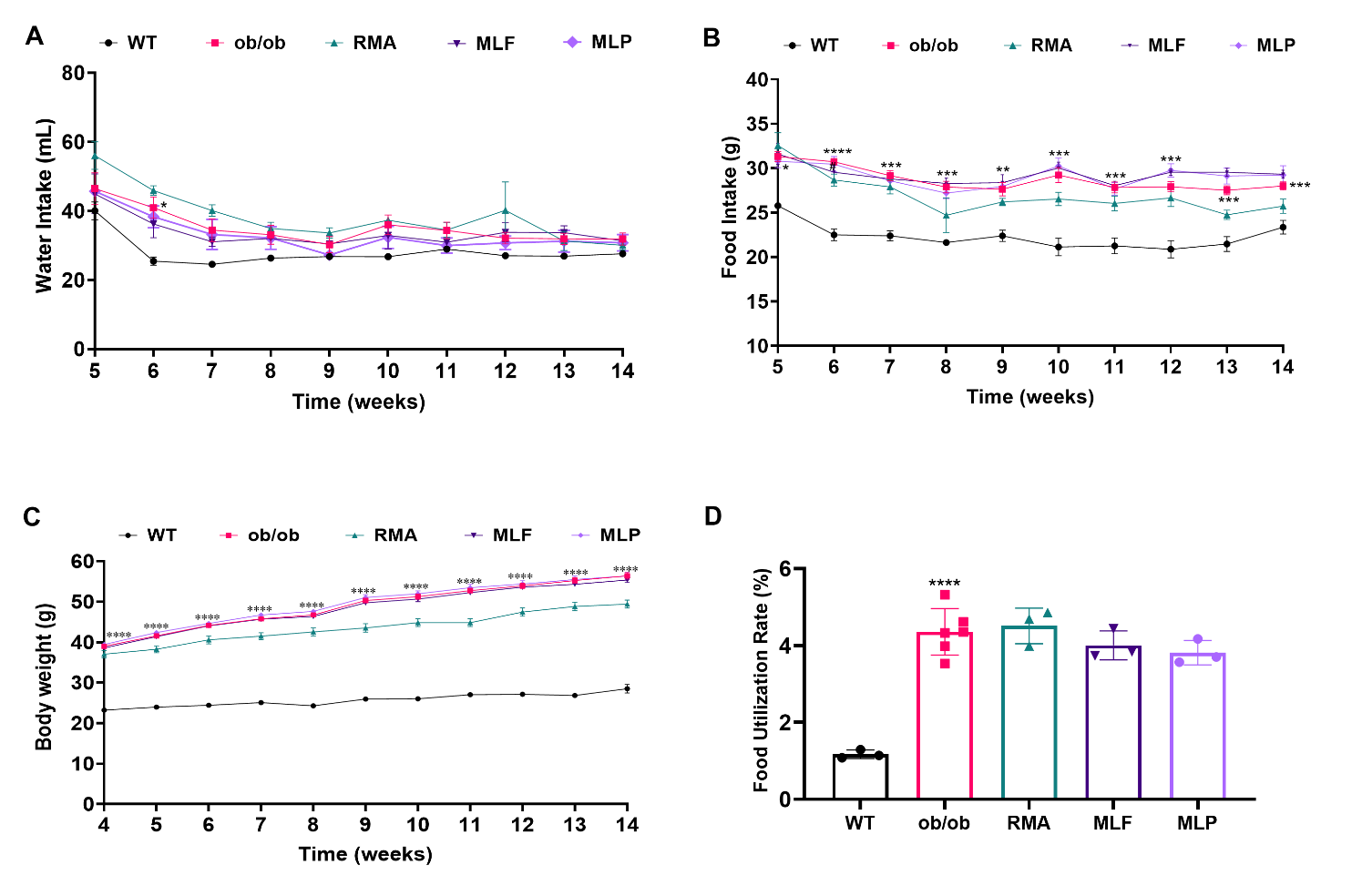
**Supplementary Figure 1.** The effects of RMA, MLF and MLP treatments on water intake, food intake, body weight and food utilization in ob/ob mice. (A)Water intake. (B) Food intake. (C) Body weight. (D) Food utilization. *P < 0.05, **P < 0.01, *** P < 0.001, ****P < 0.0001, compared with WT group.


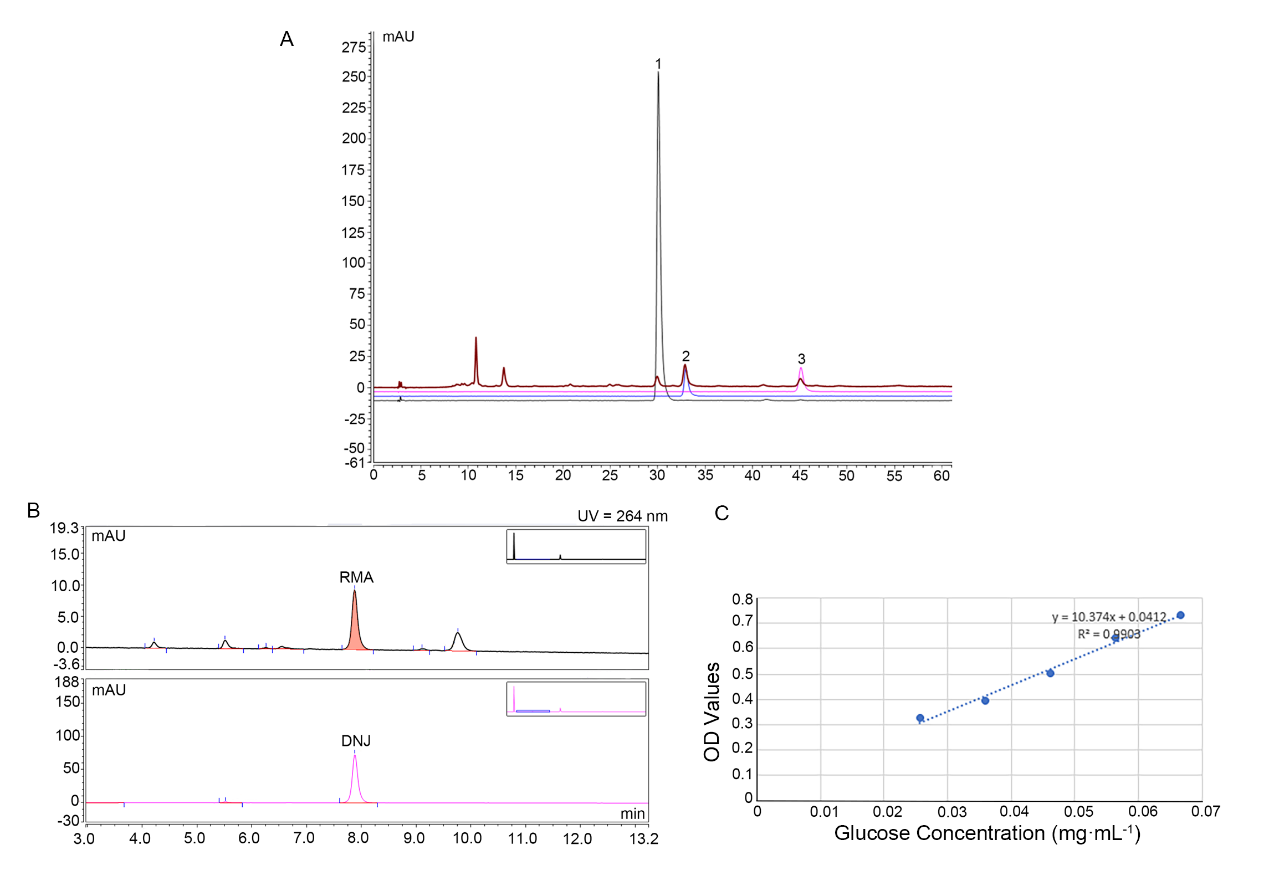


**Supplementary Figure 2.** Glucose linear standard curve from the phenol‒sulfuric acid assay.

## Supplementary Tables

**Supplementary Table 1.** Elution gradient of MLF.

| Time(min) | A (%) | B (%) |
| --- | --- | --- |
| 0 | 95 | 5 |
| 5 | 88 | 12 |
| 11 | 88 | 13 |
| 21 | 85 | 15 |
| 22 | 85 | 15 |
| 32 | 84 | 16 |
| 39 | 84 | 16 |
| 41 | 83 | 17 |
| 50 | 83 | 17 |
| 51 | 82 | 18 |
| 61 | 82 | 18 |
| 69 | 79 | 21 |
| 73 | 79 | 21 |
| 77 | 78 | 22 |
| 78 | 78 | 22 |
| 86 | 72 | 28 |
| 88 | 68 | 32 |
| 92 | 65 | 35 |
| 102 | 62 | 38 |

**Supplementary Table 2.** Elution gradient of RMA.

| Time(min) | A (%) | B (%) |
| --- | --- | --- |
| 0 | 25 | 75 |
| 22 | 52.5 | 47.5 |
| 23 | 62.5 | 37.5 |
| 38 | 62.5 | 37.5 |
| 39 | 25 | 75 |
| 43 | 25 | 75 |

**Supplementary Table 3.** Sequences of polymerase chain reaction primers used in the present study.

| Gene | GenBank accession no. | | Primer sequence |
| --- | --- | --- | --- |
| *GAPDH* | NM_001256799.3 | F: AATCCCATCACCATCTTCCAG  R: AAATGAGCCCCAGCCTTC | |
| *ADORA1* | NM_000674.3 | F: TTGGCTGGAACAATCTGAGTG  R: TGCTTGCGGATTAGGTAGAAGA | |
| *PPARG* | NM_001171818.2 | F: ATACATAAAGTCCTTCCCGCTG  R: GGCGGTTGATTTGTCTGTTG | |
